# Target discovery of acivicin in cancer cells elucidates its mechanism of growth inhibition†
†Electronic supplementary information (ESI) available: Synthesis, cloning, protein expression, purification and biochemical assays. See DOI: 10.1039/c4sc02339k



**DOI:** 10.1039/c4sc02339k

**Published:** 2014-09-16

**Authors:** Johannes Kreuzer, Nina C. Bach, Daniel Forler, Stephan A. Sieber

**Affiliations:** a Center for Integrated Protein Science CIPSM , Institute of Advanced Studies IAS , Department Chemie , Lehrstuhl für Organische Chemie II , Technische Universität München , Lichtenbergstrasse 4 , 85747 Garching , Germany . Email: stephan.sieber@tum.de ; Fax: +49 8928913210 ; Tel: +49 8928913302; b Bayer HealthCare Bayer Pharma AG , Müllerstr. 178 , 13353 Berlin , Germany

## Abstract

Acivicin is a natural product with diverse biological activities. Several decades ago its clinical application in cancer treatment was explored but failed due to unacceptable toxicity. The causes behind the desired and undesired biological effects have never been elucidated and only limited information about acivicin-specific targets is available. In order to elucidate the target spectrum of acivicin in more detail we prepared functionalized derivatives and applied them for activity based proteomic profiling (ABPP) in intact cancer cells. Target deconvolution by quantitative mass spectrometry (MS) revealed a preference for specific aldehyde dehydrogenases. Further in depth target validation confirmed that acivicin inhibits ALDH4A1 activity by binding to the catalytic site. In accordance with this, downregulation of ALDH4A1 by siRNA resulted in a severe inhibition of cell growth and might thus provide an explanation for the cytotoxic effects of acivicin.

## Abstract 2

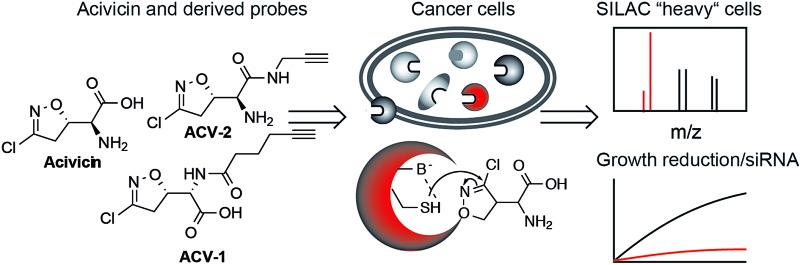
Using a chemical proteomic strategy we analyzed the targets of acivicin and provided a mechanistic explanation for its inhibition of cancer cell growth.

## Introduction

**Fig. 1:**
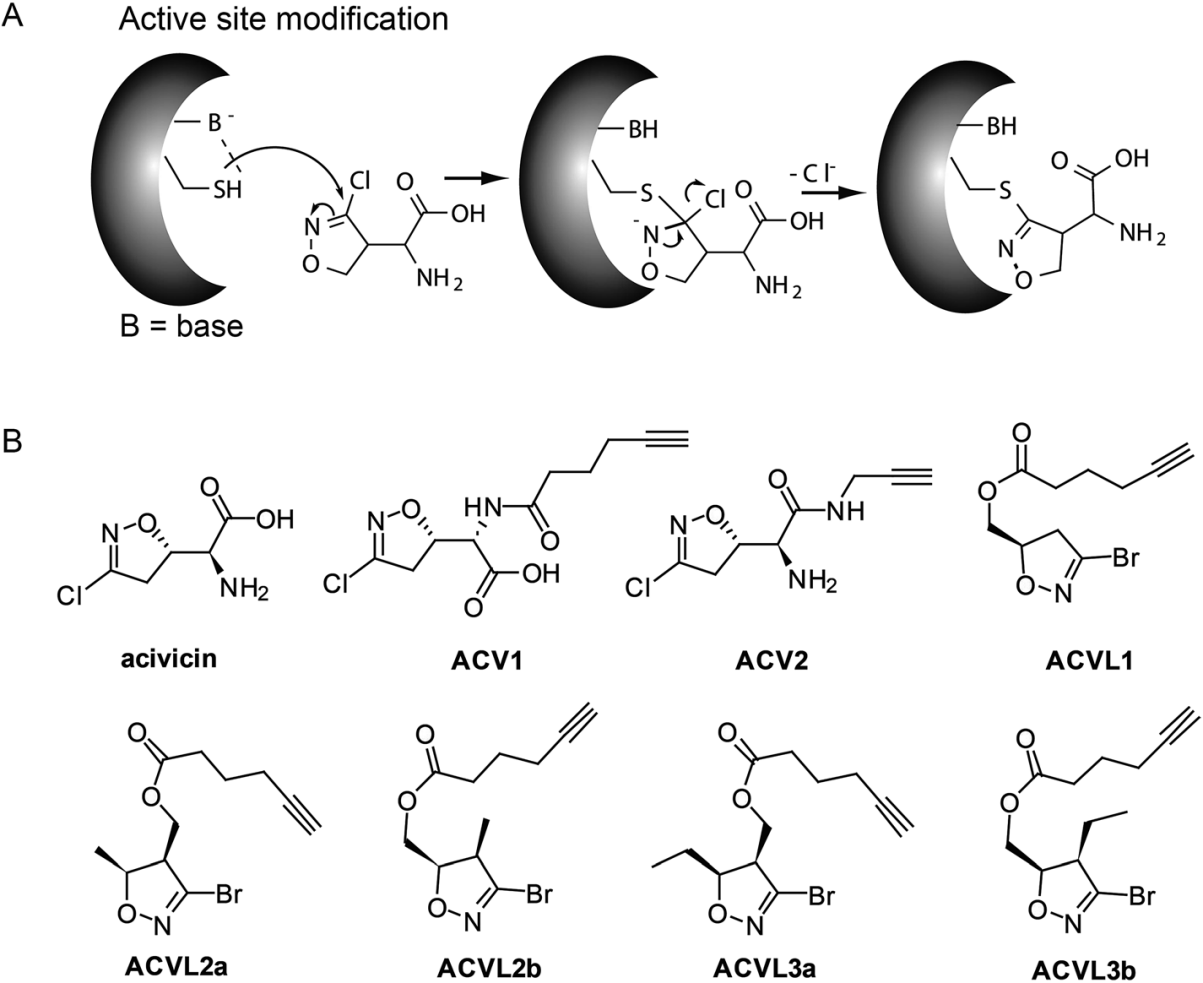
Mechanism of inhibition by acivicin through direct modification of the active site nucleophile, *e.g.* cysteine (A) and structural overview of acivicin and its derivatives used in this study (B).

Acivicin (ACV) is a natural product produced by *Streptomyces sviceus* that exhibits a diverse set of biological activities ranging from anti-cancer to anti-parasitic properties (Fig. 1). Since its discovery in 1972 ACV has been extensively studied for its putative application as an anti-tumor drug.^1–3^ Initial target predictions suggested that the structure of ACV mimics that of the natural amino acid glutamine and thus may lead to the inhibition of associated pathways. *In vitro* studies confirmed the inactivation of several glutamine dependent amidotransferases, including CTP synthase, carbamoyl phosphate synthetase II and XMP aminase that are involved in purine and pyrimidine metabolism.^4–11^ Corresponding decreases in cellular CTP and GTP levels were observed.^6^ Based on these properties ACV was evaluated for cancer therapy and progressed into several clinical trials. However, due to the occurrence of severe neurotoxicity ACV could not be approved as a drug.^12–16^ A structural relationship to the neurotoxic agents ibotenic acid and muscimol could provide an explanation for the side effects and points towards metabolic conversion of ACV into a toxic species in the organism (ESI Scheme 1†).

The target characterization of acivicin dates back into the early 1980s. While no global proteome study in human cells for target and off-target decoding has been conducted to date, it remains to be seen whether the amidotransferases, which were characterized *in vitro*, are the relevant biological targets. For example, although co-crystallization of ACV with bacterial gamma-glutamyltranspeptidase (gGT) revealed specific binding,^17–19^ a rather weak IC_50_ of 0.3 mM was observed for the inhibition of bovine gGT. Similarly, full inactivation of the human enzyme was observed at a concentration of 0.45 mM.^4,20^ Moreover, no significant gGT dependent response could be obtained in a cellular apoptosis model.^21^ Although these results suggest that ACV does not exert its clinical effects through the inhibition of human gGT, it is still advertised as a broadly applicable gGT inhibitor.

We recently investigated the bacterial targets of ACV-inspired 4-chloro- and 4-bromo-isoxazole probes by a proteome wide scan and obtained a clear preference of these compounds to irreversibly react with the active site cysteine of aldehyde dehydrogenases (ALDH).^22^ Small variations in the compound side chain length and position changed the binding preferences for individual enzymes. In order to investigate the native interactions between ACV and protein targets in cancer cells we extended our previous approach and synthesized two novel probes (ACV1, ACV2) that contain an alkyne handle and closely mimic the natural product. In combination with a selection of our previously synthesized small molecule probe collection (Fig. 1) we investigated probe cytotoxicity in HepG2 cells. Subsequent target identification of the molecules revealed a strong preference for a specific subset of ALDHs. Among those, ALDH4A1 was confirmed as an unprecedented target of ACV and the relevance of this enzyme for cellular viability demonstrated by its downregulation *via* siRNA studies. In addition we identified carboxylesterase 1 (CES1) as the target of a probe derived metabolite.

## Results and discussion

### Design and synthesis

ACV represents a small molecule with a conserved 4-chloroisoxazole motif. This core is electrophilic and reacts according to an addition–elimination mechanism with nucleophilic serine or cysteine active sites by displacement of the chlorine atom (Fig. 1A). Previous attempts to investigate the structure–activity-relationship (SAR) of acivicin suggested several restrictions. While conservative structural alterations, *e.g.* the replacement of chlorine with bromine, were tolerated, constrained analogs were inactive.^23,24^ In order to investigate the cellular targets of acivicin we selected the free carboxylic acid as well as the free amine as two potential modification sites for the attachment of an alkyne handle (ESI Scheme 2†). The alkyne represents a benign tag for bio-orthogonal modifications *via* the Huisgen–Sharpless–Meldal click reaction for the incorporation of fluorescent or affinity tags that are required to visualize and identify targets, respectively.^25–27^ Both probes were synthesized by the reaction of commercially available acivicin with hexynoic acid (ACV1) or propargyl amine (ACV2) by standard peptide coupling procedures (ESI Scheme 2†). The resulting alkyne probes were purified by HPLC and subsequently tested for biological activity.

### HepG2 cell growth inhibition with ACV derivatives

**Fig. 2:**
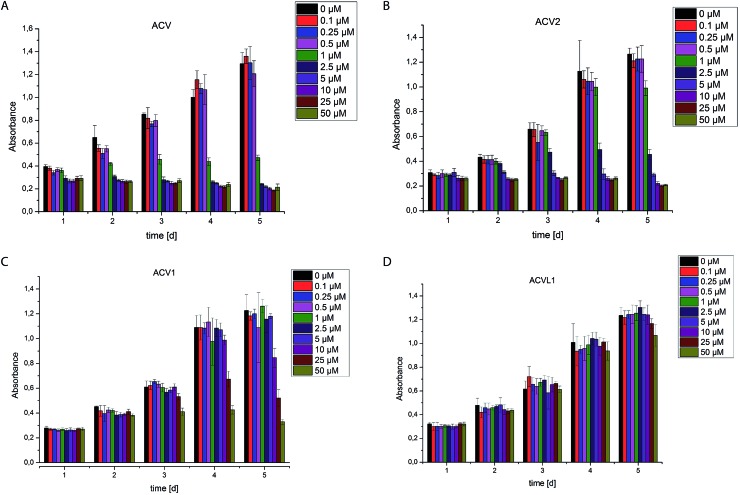
Cell growth of HepG2 cells in the presence of different concentrations of compounds ACV (A), ACV2 (B), ACV1 (C) and ACVL1 (D). Experiments were carried out five times for each concentration in at least two independent experiments. Error bars display the standard deviation of the mean.

The two novel ACV probes together with five 4-bromo-isoxazole compounds from a previous study^22^ (Fig. 1) were tested for cell growth inhibition of human HepG2 cells (hepatocellular carcinoma). Cell growth was monitored by crystal violet staining for several days in the presence of varying concentrations of compounds. While the 4-bromo-isoxazole compound ACVL1 did not reveal any growth inhibition, ACV1 significantly reduced cellular growth after five days with an IC_50_ of 14 μM (Fig. 2). ACV2 already significantly inhibited cell growth after two days. The IC_50_ after five days decreased to 1.6 μM which is about 2-fold higher than the IC_50_ of unmodified ACV (0.7 μM) (Fig. 2 and ESI Fig. 1†). This result is in agreement with the IC_50_ reported for ACV on rat hepatoma cells (0.5 μM after 7 days).^28^

### Target decoding in murine liver tissue and human liver cell culture

Based on cell toxicity studies (for bioactivity of ACVL2a and ACVL2b, see ESI Fig. 2†) three groups of important chemical tool compounds were discovered: (i) compounds that contain the privileged 4-bromo-isoxazole motif but do not exhibit any bioactivity (ACVL1, ACVL2a, ACVL2b), (ii) ACV1 with moderate activity and (iii) ACV2 with comparable activity to ACV. Since all chemical tool compounds are equipped with an alkyne handle their irreversible targets can be directly identified by activity based protein profiling (ABPP) (Fig. 1).^29–33^ Differences in the labeling pattern between the three groups of probes should help to rank and correlate the identified proteins with regards to their biological relevance.

**Fig. 3:**
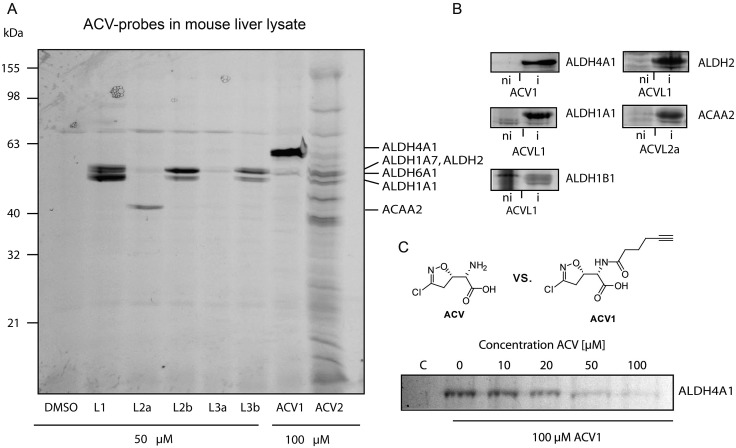
Protein targets of Acivicin-derived probes. (A) Fluorescent SDS-PAGE of labeling in mouse liver lysate. Proteins identified *via* mass spectrometry are indicated on the right-hand side. Please refer to ESI Table 2† for abbreviations. (B) Confirmation of identified target proteins by overexpression and labeling with the different probes in *E. coli*. Labeling was performed at probe concentrations of 50 μM for 2 h in intact cells. Fluorescence gels compare the labeling signals under non-induced (ni) *versus* induced (i) protein expression conditions. (C) Competitive labeling of ALDH4A1 with ACV1 and acivicin in HepG2 lysate. The fluorescence gel shows decreasing ACV1 signal with increasing amounts of pre-incubated acivicin that disappears at equimolar concentrations of both substances.

We initiated the target analysis in mouse liver lysates since metabolic enzymes including amidotransferases are abundant in this tissue and thus this may represent a good system to optimize probe handling before moving onto living cells. The profiling was carried out with a collection of seven probes that comprise members of the three classes. Each probe was incubated with tissue lysate for one hour. The subsequent click reaction with rhodamine azide was followed by SDS-PAGE analysis and fluorescent scanning. First, we varied the concentration of probe and determined optimal conditions. Labeling of specific bands could be achieved at concentrations as low as 1 μM for ACV1 (ESI Fig. 3†). Concentrations of 50–100 μM were sufficient for saturated labeling. Second, we used this optimal concentration range for all probes and compared their labeling pattern (Fig. 3).

Interestingly, only a limited number of targets with good signal to noise ratio were labeled. 4-Bromo-isoxazole probe ACVL2b revealed a preference for several proteins around 50 kDa. Preparative enrichment with biotin–rhodamine–azide and mass spectrometric analysis of these bands revealed their identity as aldehyde dehydrogenases ALDH1A1, ALDH1A7 and ALDH2 (ESI Tables 1 and 2†). In addition, the single band derived from probe ACVL2a was assigned to an enzyme involved in fatty acid metabolism, 3-ketoacyl-CoA thiolase. ACV1 showed a strong preference for ALDH4A1 and also labeled ALDH6A1 to a minor extent. ACV2 was less selective and bound to several proteins including the ones mentioned above. Although these pre-screen results revealed covalent binding of acivicin derivatives to the family of aldehyde dehydrogenases we could not identify a single amidotransferase, the putative targets described previously. This could be due to the modified ACV scaffold or abundances below our detection limit.

In order to investigate the target preferences of unmodified ACV we pre-incubated the lysate with an excess of natural product ACV and subsequently added several probes to visualize enzymes with an accessible (not ACV blocked) active site. Interestingly, ALDH4A1 did not bind to the ACV1 probe after ACV pre-incubation even at an equimolar ratio, suggesting a high affinity interaction of this protein with the natural product (ESI Fig. 4†). In contrast, several ACVL1 targets including ALDH1A1 either did not disappear at all or only disappeared at a much higher excess of ACV.

We next expanded our studies to living HepG2 cells derived from human liver cancer tissue. *In situ* labeling of intact HepG2 may reveal a different signature of putative targets and also enables quantitative MS analysis *via* stable isotope labeling of amino acids in cell culture (SILAC).^34^ HepG2 cells were incubated with probes (50–100 μM) in PBS for two hours. After removal of excess probe the cells were lysed, proteins modified by click chemistry and the fluorescent protein pattern of the soluble fraction analyzed as described above. Interestingly, the fluorescent profile was similar to the one observed in mouse liver lysate (Fig. 3 and ESI Fig. 5†).

Labeling was generally performed in PBS and not media since it is known that acivicin competes with amino acids for cellular uptake *via* transporters.^35,36^ As expected, in media elevated concentrations (200 μM) and incubation times (3.5 h) were required to reveal comparable signals with only slightly reduced intensity (ESI Fig. 6†).

To unravel the identity of these target proteins we performed MS identification of the gel bands. In addition to the label-free identification of protein targets, we cultivated HepG2 cells in media containing heavy isotope-labeled arginine and lysine (ESI Fig. 7†). The heavy labeled cell population was treated with probes (50–100 μM) in PBS for two hours. The medium labeled cell population was treated with DMSO under the same conditions as control. Both samples were lysed, adjusted to the same protein concentration, mixed, clicked, enriched and finally separated on SDS-PAGE for fluorescent imaging. Isolation and MS-analysis of gel bands revealed significant SILAC enrichment factors (>2) of probe treated samples for a similar set of aldehyde dehydrogenases to those obtained in the mouse liver (ESI Tables 1, 3 and 4†). In order to focus the target analysis on unmodified ACV, we performed a separate MS-based experiment in which we pre-treated medium labeled cells with 100 μM of unmodified ACV and subsequently added 100 μM ACV1 probe. To exclude influences of the different isotope variants of amino acids on the outcome of the experiment, one experiment was carried out where heavy labeled cells were pre-treated with 100 μM ACV and incubated with 100 μM ACV1 probe (label switch). Interestingly, ALDH4A1 was the most sensitive to this competition (ESI Tables 1 and 5†). This is in agreement with gel based experiments were an equimolar ratio was sufficient to almost completely remove the probe signal (Fig. 3C and ESI Fig. 8†). These results thus support a specific interaction of ALDH4A1 with the natural product. In contrast, ALDH1A1 again appeared to be a probe specific target as its labeling was not reduced in the presence of excess ACV (ESI Fig. 9†).

As labeling in PBS does not reflect the conditions of the growth inhibition experiments (Fig. 2) we investigated the cell permeability and target binding of the most potent probes ACV1 and ACV2 under cell culture conditions with incubation of up to several days. HepG2 cells were cultured in RPMI media and treated with 25 μM ACV1 and 10 μM ACV2, concentrations close to the inhibition of cell growth. Targets that bind under these conditions are promising candidates for a detailed mechanism of action analysis.

For ACV1 a single target was observed that reproducibly changed its localization over time into the insoluble fraction, indicating that probe binding altered the protein physical properties. As MS identification of the insoluble protein proved difficult, we confirmed that this protein was ALDH4A1 *via* a comparison of probe-derived fluorescence and protein-specific immunostaining on the same membrane (ESI Fig. 10†).^37^

**Fig. 4:**
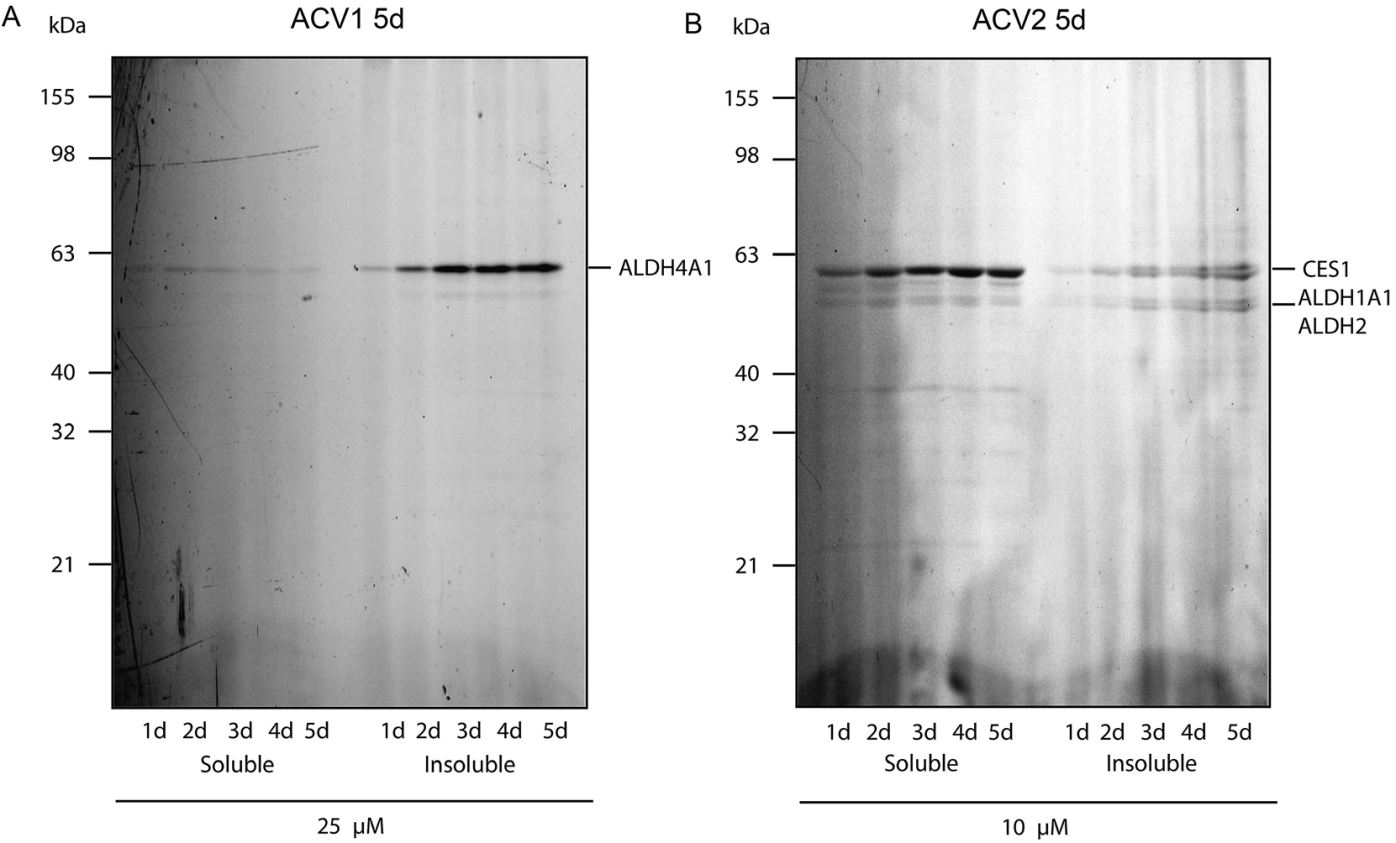
Fluorescence scan of HepG2 cells after probe incubation for several days. (A) ACV1 and (B) ACV2. Target identities are indicated on the right.

Interestingly, SDS-gel analysis of ACV2 labeling revealed one strong fluorescent band that exhibited a maximum intensity after five days of incubation (Fig. 4). In addition only a few weaker bands were visible suggesting that ACV2 selectivity increases at concentrations close to its IC_50_ compared to the higher concentrations used in the initial studies above. MS based target identification revealed ALDH1A1 and ALDH2 as the most likely hits of the weak bands. Surprisingly, the strong upper band was identified as carboxylesterase 1 (CES1) (ESI Tables 1 and 6†). CES1 is a major hydrolase in the human liver that is involved in the metabolism of several drugs and endogenous compounds. However, the exact role of CES1 in metabolic control is largely unknown.^38,39^ To exclude that ALDH4A1 and CES1 expression was artificially induced and elevated in response to probe incubation, we confirmed their steady expression levels by Western Blot analysis over five days (ESI Fig. 11†).

### Target overexpression and inhibition

In order to investigate the identified ALDHs in more detail we cloned the corresponding genes in vectors and recombinantly overexpressed the enzymes. For CES1 commercially available recombinant protein was used. Incubation of the probes with cells expressing the proteins *in situ* confirmed target binding in case of the ALDHs (Fig. 3B). Surprisingly, CES1 spiked into A549 lysate was not labeled by ACV2; however, a serine hydrolase-specific fluorophosphonate (FP) probe bound to the enzyme, suggesting that it was functionally active (ESI Fig. 12†).

**Fig. 5:**
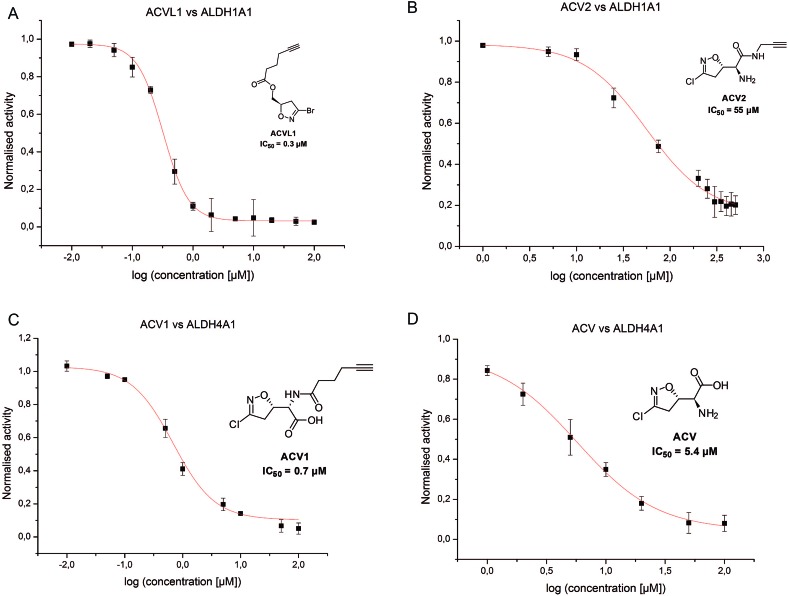
IC_50_ measurements for the compounds ACVL1 (A), ACV2 (B), ACV1 (C) and ACV2 (D) for the dehydrogenases ALDH1A1 (A and B) and ALDH4A1 (C andD). Each compound was tested in two independent trials in triplicates.

The putative target proteins were purified and inhibition assays with the compounds performed. ALDH1A1 and ALDH4A1 were incubated with ACVL1, ACV1, ACV2 and ACV for 30 min and the turnover was monitored for another 45 min. As all ACV derivatives are covalent and thus time dependent inhibitors we compared their IC_50_ values after the same incubation time. While ALDH1A1 was inhibited by ACVL1 (IC_50_ = 0.3 μM), ACV2 exhibited a higher IC_50_ value of 55 μM (Fig. 5). ACV did not inhibit the enzyme at all, which is in line with the *in situ* competition experiments and demonstrates that ALDH1A1 is not a target of the natural product (ESI Fig. 13†). Interestingly, ALDH4A1 was inhibited by ACV1 and ACV, with IC_50_s of 0.7 μM and 5.4 μM respectively (Fig. 5). A mutation of the ALDH4A1 catalytic Cys348 to Ala as well as pre-incubation of the native enzyme with the ALDH inhibitor disulfiram resulted in a lack of labeling thus demonstrating that the probe is active site (Cys348) directed (ESI Fig. 14†). Considering the competition observed with ACV in the ALDH4A1 *in situ* experiments, the IC_50_ values support the assignment of this enzyme as a target of the natural product. In contrast, ACV2 did not show ALDH4A1 inhibition (ESI Fig. 13†), further highlighting a tight SAR within this enzyme family. These results also demonstrate that neither ACV1 nor ACV2 addresses the full target spectrum of unmodified ACV, and emphasizes the value of utilizing both probes.

**Fig. 6:**
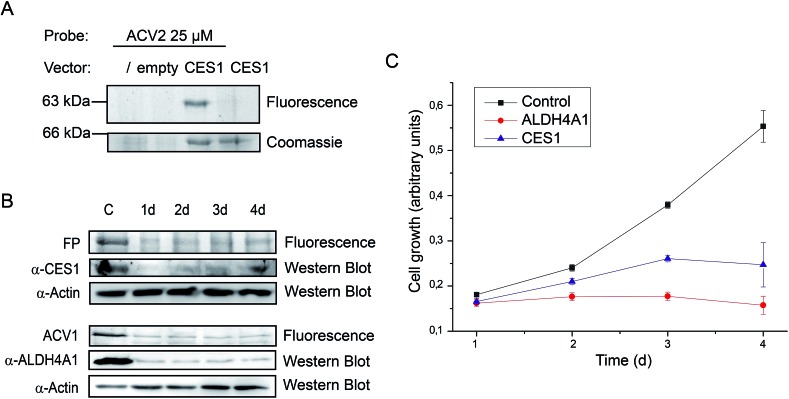
Biological impact of the ACV1 and ACV2 targets ALDH4A1 and CES1. (A) Labeling with ACV2 in living cells containing a vector for expression of CES1 (empty = vector without CES1 gene). (B) siRNA knock-down studies of CES1 and ALDH4A1 in HepG2 cells over a time range of 4 days after transfection monitored by western blot. α-CES1 = anti-CES1 antibody, α-actin = anti-actin antibody. (C) Cell growth of ALDH4A1 and CES1 siRNA transfected cells in comparison to control cells only treated with transfection reagent over four days. Each time point was carried out five times in 3 independent experiments. Error bars display the standard deviation of the mean.

Finally, we followed up on CES1 as putative hit. Recombinant CES1 was incubated with ACV and ACV2 and the turnover was monitored for 30 min. No inhibition with ACV2 and ACV was observed (ESI Fig. 13†). As no direct binding to ACV2 was obtained, the results suggest cellular metabolism of the probe into a derivative with CES1 affinity. This could also explain the delay in labeling which required at least one day of incubation (Fig. 4). We thus incubated recombinant CES1 with ACV2 for one day but did not obtain any binding suggesting that CES1 alone is not sufficient for metabolite formation. In addition, spiking of CES1 into an A549 lysate did not lead to ACV2 conversion and binding (ESI Fig. 15†) indicating that the intact cell is required to metabolize ACV2. To analyze CES1 labeling in intact cells independent of endogenous expression levels, we transiently overexpressed CES1 for three days in HEK293T cells, which possess a very low endogenous CES1 level, and added ACV2 one day after transfection. In contrast to cells that were not transfected or transfected with an empty vector, the CES1 labeled band could clearly be detected in the cells containing CES1 plasmid, hence confirming CES1 as an ACV2 target in living cells (Fig. 6A and ESI Fig. 16†). Mutation of the active site Ser221 to Ala resulted in a lack of labeling, confirming that the inhibitor is active site directed (ESI Fig. 14†).

### Target validation by siRNA knockdown studies

Target identification and subsequent inhibition studies had revealed unprecedented binding of ACV to ALDH4A1. Furthermore, in living cells CES1 was identified as the primary target of an unknown metabolically modified ACV2 derivative. To investigate the functional roles of ALDH4A1 and CES1 in cellular viability we performed siRNA knockdown studies. siRNA transfected HepG2 cells showed diminished protein expression after one day. Protein levels reached their minimum on days 2 to 4 and 3 to 4 after transfection for CES1 and ALDH4A1, respectively (Fig. 6B). Analysis of cell growth over the same time range revealed diminished growth of cell populations transfected with CES1 and ALDH4A1 siRNA in comparison to cells only treated with transfection reagent, demonstrating that both targets are important for cell growth (Fig. 6C). The importance of the validated target ALDH4A1 for cell growth is a possible explanation for the known cytotoxicity of ACV in human cells. CES1, on the other hand, may be a specific target of ACV2.

## Conclusion

Previous studies with ACV derived probes in bacteria revealed a preference of this reactive scaffold for the aldehyde dehydrogenase enzyme family.^22^ This family of enzymes plays important roles in metabolism, detoxification, cell proliferation and cancer. They catalyze the oxidation of aldehydes to their corresponding carboxylic acids.^40–42^ ACV is described as an anti-cancer compound targeting amidotransferases and especially gGT in eukaryotic cells. However, previous studies demonstrated that ACV has a very low affinity for human gGT, suggesting that additional targets may exist contributing to the biological activity.^2^ Here, we have analyzed the covalent interaction partners of ACV *via* comparative studies with several related probe scaffolds. As in bacterial proteomes, a set of ALDH enzymes was identified *via* MS analysis. Some of these ALDHs, however, could not be confirmed as binders of the natural product ACV, suggesting that structural perturbations directed the binding of the probes to ACV other enzymes. Interestingly, we were able to confirm ALDH4A1 as a previously unknown ACV target that is furthermore important for cellular viability. ALDH4A1, also termed delta-1-pyrroline-5-carboxylate dehydrogenase, participates in glutamate synthesis by converting 1-pyrroline-5-carboxylate, a substrate with high structural similarity to ACV (ESI Scheme 3†).^43^ It has been proposed that during enzyme catalysis the active site Cys348 carries out a nucleophilic attack on the aldehyde group to form a hemithioacetal which is further oxidized to glutamate in a NAD+ dependent process.^44^ ACV is therefore likely a substrate mimic that inactivates the enzyme.

There are 19 isoforms of human aldehyde dehydrogenases that are involved in various cellular metabolic processes essential for numerous physiological, pathological and pharmacological processes. Recent studies have revealed that elevated activity of various ALDHs is a hallmark of cancer stem cells and certain tumors.^45,46^ Moreover, correlations between ALDH expression and proliferation have been observed in normal and cancer cells as well.^45,47–49^ This offers an intriguing explanation for the observed bioactivity of acivicin and offers an opportunity to study the activity of selected enzymes using the diverse set of probes presented here.

We cannot exclude that cellular metabolism converts ACV into derivatives that exhibit very different properties and also address additional targets. Future studies will thus focus on ACV metabolism and corresponding target interactions.

## Methods

### Cell growth assay

HepG2 cells from subconfluent cultures were used for the assay. Specifically, 6000 cells per well were seeded in 96 well flat-bottom plates (*Nunclon*) in 100 μL medium and cultured for 12 h with one plate for each time interval. Compounds were diluted 1 : 100 from 100× DMSO stocks in 100 μL of the appropriate culture medium and added to the cells after careful removal of the culture medium without compounds. After incubation (24 h, 48 h, 72 h, 96 h and 120 h) 10 μL of 11% glutaraldehyde solution was added to each well and the plate was incubated at room temperature for 30 min. Media containing glutaraldehyde was removed and cells were washed 10 times with ddH_2_O and dried overnight. After fixation cells were stained by adding 100 μL of 0.1% crystal violet solution in water to each well followed by a 30 min incubation step at room temperature. Crystal violet solution was removed and the wells were washed 10 times with ddH_2_O and dried over night. Next 100 μL of 10% acetic acid was added to each well and incubated at room temperature to dissolve the crystal violet. The optical density at 590 nm was measured using a *TECAN* Infinite 200pro plate reader. All measurements were performed five times and in at least three independent experiments. Error bars were calculated from standard deviation from the mean. IC_50_ values were calculated from curve fittings by Origin Pro 8.5 (*OriginLab Corporation*).

### Preparative *in situ* ABPP labeling experiments

Cells were grown to 70% confluency in Petri dishes (150 mm). The medium was aspirated and cells were washed with 10 mL PBS and then harvested in 20 mL fresh PBS by scraping. Cells were washed (800 g, 5 min), resuspended in 1000 μL PBS containing probes at the appropriate concentration and incubated for 2 hours at RT. Subsequently, cells were spun for 5 min at 800 g at RT to remove PBS with excess of probe, washed twice with 500 μL PBS and resuspended in 500 μL lysis buffer (PBS, 1% NP-40, 1% DOC). Soluble and insoluble fraction were separated by centrifugation at 14 800 rpm for 60 min at 4 °C. Insoluble pellets were resuspended in 500 μL lysis buffer by sonication under ice cooling. Protein concentration was assayed (Rotiquant universal, *Carl Roth Laborbedarf*) and adjusted to 2 mg mL^–1^ in PBS.

### Click reaction and preparative gel-based analysis

To 947 μL proteome solution 3 μL trifunctional linker^50^ (10 mM in DMSO) was added, followed by 10 μL TCEP solution (53 mM in ddH_2_O) and 30 μL ligand TBTA (83 mM in DMSO/*tert*-butanol). The samples were gently vortexed and the reaction was initiated by the addition of 10 μL CuSO_4_ solution (50 mM in ddH_2_O). The reaction was allowed to proceed for 1 h at RT. Reactions for enrichment were carried out together with a control lacking the probe to compare the results of the biotin–avidin enriched samples with the background of nonspecific protein binding on avidin–agarose beads. The proteins were precipitated by addition of 4 volumes of cold acetone (4 mL, –20 °C) followed by an incubation for 18 h at –20 °C. Then the proteins were pelleted (30 min, 21 000 g, 4 °C) and the supernatant was discarded. The proteins were washed with pre-chilled methanol (2 × 200 μL, –20 °C) and resuspended by sonication (5–10 s, 10% max. intensity; 15 min, 21 000 g, 4 °C). Subsequently, the pellet was dissolved at RT in 1 mL 0.2% SDS in PBS by sonication and incubated under gentle mixing with 50 μL of prewashed (3 × 1 mL 0.2% SDS in PBS) avidin–agarose beads (avidin–agarose from egg white, 1.1 mg mL^–1^ in aqueous glycerol suspension, *Sigma-Aldrich*) for 2 h at RT. The beads were washed with 0.2% SDS in PBS (3 × 1 mL), 6 M urea (2 × 1 mL) and PBS (3 × 1 mL). 50 μL of 2× SDS loading buffer was added and the proteins were released for preparative SDS-PAGE by incubation for 6 min at 96 °C. The beads were pelletized (3 min, 21 000 g) and the supernatant was isolated and stored at –80 °C. The supernatant was applied on a preparative gel, and run for 4–5 h (300 V). After gel electrophoresis, the bands were visualized using a *Fujifilm* Las-4000 luminescent image analyser containing a VRF43LMD3 lens and a 575DF20 filter. The observed bands were excised and diced into pieces of approximately 1 mm length, prior to further processing (see SI†).
