# Supplementary material for: Target discovery of acivicin in cancer cells elucidates its mechanism of growth inhibition
Source: Chem Sci. 2014 Sep 16;6(1):237–45. doi: 10.1039/c4sc02339k (PMC4285139; doi:10.1039/c4sc02339k)
Supplement: Supplementary file 6 [file SC-006-C4SC02339K-s006.pdf]

Electronic Supplementary Material (ESI) for Chemical Science.
This journal is © The Royal Society of Chemistry 2014

Supporting Information

Target discovery of acivicin in cancer cells elucidates its mechanism of growth inhibition

Johannes Kreuzer, Nina C. Bach, Daniel Forler and Stephan A. Sieber

Materials

All chemicals used were of reagent grade or higher and used without further purification. Solvents for reactions were of HPLC-grade and purchased from *Sigma Aldrich*, *Bachem* or *Acros*. Solvents for chromatography and workup purposes were generally of reagent grade and purified before use by distillation. Acivicin was purchased from *Santa Cruz Biotechnology* (*SCBT*). FP-probe was custom synthesized by *SYNCOM Corp.*, Groningen, NL; (Syncom #17682) as already described before.[1] ACVL probes were synthesized as described before.[2] Recombinant CES1 was purchased from *Sigma Aldrich*.

^{1}H- and ^{13}C-NMR spectra were recorded on a *Bruker Avance I 360* (360 MHz), a *Bruker Avance I* (500 MHz) or a *Bruker Avance III 500* (500 MHz) NMR-System and referenced to the residual proton and carbon signal of the deuterated solvent, respectively.
HR-ESI-MS, HR-LC-ESI-MS, HR-APCI-MS and HR-LC-APCI-MS mass spectra were recorded with a *Thermo Finnigan LTQ FT Ultra* coupled with a *Dionex UltiMate 3000* HPLC system. ESI-MS and LC-ESI-MS mass spectra were recorded with a *Thermo Finnigan LCQ ultrafleet* coupled with a *Dionex UltiMate 3000* HPLC system.
HPLC analysis was accomplished with a *Waters 2695 separations module*, an *X-Bridge™ C18 3.5 µm OBD™* column (4.6 x 100 mm) and a *Waters 2996 PDA detector*.
HPLC separation was accomplished with a *Waters 2545 quaternary gradient module*, an *X-Bridge™ Prep C18 10 µm OBD™* (50 x 250 mm), an *X-Bridge™ Prep C18 5 µm OBD™* (30 x 150 mm) or an *YMC Triart C18 5 µm* column (10 x 250 mm), a *Waters 2998 PDA detector* and a *Waters Fraction Collector III*.

2,5-Dioxopyrrolidin-1-yl hex-5-ynoate (2)

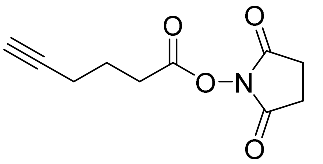

2.21 mL 5-hexynoic acid (20 mmol, 1 eq.) and 2.42 mL *N*-hydroxysuccinimide (21 mmol, 1.05 eq.) were dissolved in 80 mL CH_2Cl_2. 4.33 g DCC (dicyclohexylcarbodiimide) (21 mmol, 1.05 eq.) were subsequently added in portions. The reaction mixture was stirred for 2 h at RT and filtered. The filtrate was washed twice with 50 mL water and the combined aqueous phases were washed with 50 mL CH_2Cl_2. The combined organic phases were dried over Na_2SO_4 and the solvent was removed under reduced pressure. The crude product was purified by column chromatography (silica gel, hexane:ethyl acetate = 6:4) to yield 1.44 g (34%) of an yellowish oil. Data is consistent with that reported in the literature.[3]

***R*f** (hexane:ethyl acetate = 6:4) = 0.53

^{1}H NMR (500 MHz, Chloroform-*d*) δ (ppm) = 2.84 (d, *J* = 5.7 Hz, 4H), 2.77 (t, *J* = 7.4 Hz, 2H), 2.35 (td, *J* = 6.9, 2.6 Hz, 2H), 2.02 (t, *J* = 2.6 Hz, 1H), 1.96 (p, *J* = 7.1 Hz, 2H).

^{13}C NMR (126 MHz, $CDCl_3$) δ (ppm) = 169.23, 168.30, 82.53, 69.97, 29.77, 25.71, 23.43, 17.71.

(S)-2-((S)-3-Chloro-4,5-dihydroisoxazol-5-yl)-2-(hex-5-ynamido)acetic acid (3, ACV1)

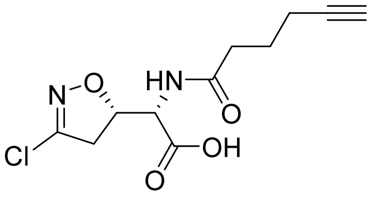

10.0 mg acivicin (0.056 mmol, 1.0 eq.) were dissolved in 600 µL ddH2O and 300 µL acetonitrile. 23.6 mg **2** (0.112 mmol, 2.0 eq.) and 4 µl diisopropylethylamine were added and the solution was stirred for 14 h at RT. The crude product was purified by HPLC (5-60% MeCN, 0.1% TFA, 15 min) to give 9.6 mg (76%) white solid.

HPLC t_R = 10.9 min (5-60% MeCN, 0.1% TFA, 15 min)

^{1}H NMR (500 MHz, Acetonitrile-*d*$_3$) δ (ppm) = 6.97 (d, *J* = 8.0 Hz, 1H), 5.06 (ddd, *J* = 11.5, 7.4, 4.6 Hz, 1H), 4.69 (dd, *J* = 8.3, 4.6 Hz, 1H), 3.39 (dd, *J* = 17.8, 11.2 Hz, 1H), 3.24 (dd, *J* = 17.8, 7.4 Hz, 1H), 2.31 (t, *J* = 7.4 Hz, 2H), 2.23 – 2.17 (m, 3H), 1.75 (p, *J* = 7.1 Hz, 2H).

^{13}C NMR (126 MHz, CD_3CN) δ (ppm) = 173.61, 170.21, 150.69, 84.53, 82.62, 70.15, 55.08, 41.32, 34.95, 25.15, 18.12.

HRMS-ESI (m/z): $C_{11}H_{14}ClN_2O_4^+$ $[M+H]^+$, calc.: 273.0636, found: 273.0636.

(S)-2-((*Tert*-butoxycarbonyl)amino)-2-((S)-3-chloro-4,5-dihydroisoxazol-5-yl)acetic acid (4)

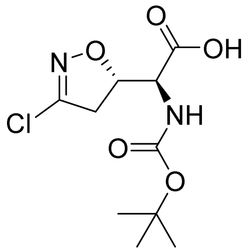

To a solution of 10.0 mg acivicin (0.056 mmol, 1.0 eq.) in 160 µL water a solution of 13.5 mg di-*tert*-butyl dicarbonate (0.061 mmol, 1.1 eq.) in 210 µL acetonitrile was added. 10 µL of triethylamine (0.072 mmol, 1.3 eq.) were added and the reaction was stirred for 14 h at RT. The solvent was removed under reduced pressure and gave 15 mg compound **4** as a yellow oil (97%). The product was used without further purification.

HPLC t_R = 13.5 min (5-60% MeCN, 0.1% TFA, 15 min)

^{1}H NMR (360 MHz, Acetonitrile-*d*$_3$) δ (ppm) = 5.75 (s, 1H), 5.14 – 5.04 (m, 1H), 4.03 (dd, *J* = 6.8, 2.8 Hz, 1H), 3.53 (dd, *J* = 17.1, 7.9 Hz, 1H), 3.23 (dd, *J* = 17.1, 11.4 Hz, 1H), 1.43 (s, 9H).

^{13}C NMR (91 MHz, CD_3CN) δ (ppm) = 171.51, 149.12, 146.79, 85.44, 78.47, 40.10, 30.47, 27.58.

***Tert*-butyl ((S)-1-((S)-3-chloro-4,5-dihydroisoxazol-5-yl)-2-oxo-2-(prop-2-yn-1-ylamino)ethyl)carbamate (5)**

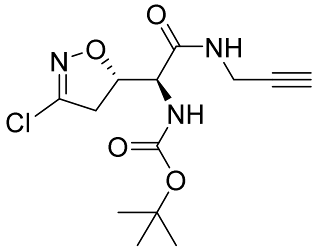

15.0 mg (0.056 mmol, 1.0 eq.) of **4** were dissolved in 50 µL acetonitrile. To this solution 24.0 mg COMU (0.056 mmol, 1.0 eq.) in 60 µL acetonitrile and 8.0 mg Oxyma Pure (0.056 mmol, 1.0 eq.) in 60 µL acetonitrile were added. Subsequently 10 µL *N*,*N*-diisopropylethylamine (0.056, 1.0 eq.) and the carboxylic acid was activated for 10 min at RT. After activation 8 µL propargylamine (0.061 mmol, 1.1 eq.) was added and the reaction mixture was stirred at RT for 1.5 h. Solvent was removed under reduced pressure and the compound was purified by HPLC (40-60% MeCN 0.1% TFA, 10 min) to give 12 mg of compound **5** (70%) as white solid.

HPLC t_R = 14.5 min (5-60% MeCN, 0.1% TFA, 15 min)

^{1}H NMR (360 MHz, Acetonitrile-*d*$_3$) δ (ppm) = 4.98 (dt, *J* = 10.8, 6.9 Hz, 1H), 4.00 – 3.92 (m, 2H), 3.34 (dd, *J* = 17.8, 10.9 Hz, 1H), 3.21 (dd, *J* = 17.8, 7.4 Hz, 1H), 2.47 (t, *J* = 2.5 Hz, 1H), 1.44 (s, 9H).

^{13}C NMR (91 MHz, CD_3CN) δ (ppm) = 169.52, 156.49, 150.61, 82.78, 80.75, 76.87, 71.96, 41.15, 29.23, 28.41.

(S)-2-amino-2-((S)-3-chloro-4,5-dihydroisoxazol-5-yl)-N-(prop-2-yn-1-yl)acetamide (6, ACV2)

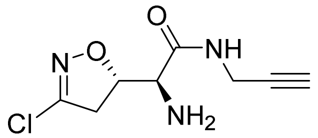

12.0 mg of **5** (0.038 mmol) were dissolved in 2.0 mL of a mixture of DCM:TFA (4:1) and stirred for 1 h. The solvent was evaporated under reduced pressure and the product was purified by HPLC (20-40% MeCN), 10 min to yield 7.0 mg of product **6** (87%) as white solid.

HPLC t_R = 6.7 min (2-50% MeCN, 10 min)

^{1}H NMR (500 MHz, Acetonitrile-d_3) δ (ppm) = 7.35 (s, 1H), 4.96 (ddd, *J* = 10.7, 8.7, 4.8 Hz, 1H), 3.94 (d, *J* = 5.8 Hz, 2H), 3.58 (d, *J* = 4.8 Hz, 1H), 3.29 – 3.14 (m, 2H), 2.43 (t, *J* = 2.5 Hz, 1H).

^{13}C NMR (126 MHz, CD_3CN) δ (ppm) = 171.95, 150.51, 84.84, 81.10, 71.64, 57.40, 39.68, 28.89.

HRMS-ESI (m/z): $C_8H_{11}ClN_3O_2^+$ $[M+H]^+$, calc.: 216.0534, found: 216.0536, δ = 0.93 ppm.

Cell culture

Cell culture media and supplements were purchased from PAA and Sigma Aldrich. HepG2 and HEK293T cells were cultivated in RPMI 1640 media supplemented with 10% fetal calf serum and 2 mM L-glutamine. A549 cells were cultivated in DMEM high glucose supplemented with 10% fetal calf serum and 2 mM L-glutamine. Cells were cultivated at 37 °C with 5% CO_2 in a Hera Cell 240i incubator (*Thermo Scientific*).

SILAC Cell culture

HepG2 cells were cultivated in Silac-RPMI-1640 media (*PAA*) supplemented with 10% dialyzed fetal calf serum and 2 mM L-glutamine. The *heavy* medium was supplemented with 0.418 mM Lysine-8 (U-13C6; U15N2, *Cambridge Isotopes*) and 0.214 mM Arg-10 (U-13C6; U15N4, *Cambridge Isotopes*). The *medium* medium was supplemented with 0.418 mM

Lysine-4 (4,4,5,5-D4, *Cambridge Isotopes*) and 0.214 mM Arg-6 (U-13C6, *Cambridge Isotopes*). HepG2 cells were cultivated until full incorporation of amino acid isotopes before carrying out experiments.

Five day labeling experiments

Analytical: Cells were grown in 6 well plates until a confluency of 70% was reached. The probes were added 1:1000 from DMSO stocks and one 1 mL media was supplemented with 1 µL DMSO as control. The media was exchanged with 1 mL media containing probes at the appropriate concentrations. After incubation (24 h, 48 h, 72 h, 96 h and 120 h) cells were washed with PBS and harvested by scraping in 1.5 mL PBS. Cells were pelleted by centrifugation at 800 g for 5 min and PBS was aspirated. Cells were lyzed by resuspension in 50 µL lysis buffer (PBS, 1% NP-40, 1% DOC) and incubation on ice for 10 min. Soluble and insoluble fraction were isolated by centrifugation at 21000 g for 20 min at 5 °C and insoluble fraction was resuspended in 50 µL lysis buffer by sonication under ice cooling. The samples then underwent *Click reaction and analytic gel-based analysis* (see below).

Preparative: Cells were grown in Petri dishes (150 mm) until they reached 70% confluency. Then the media was exchanged with 10 mL media with appropriate probe concentration and media lacking the probe as control. After incubation (24 h, 48 h, 72 h, 96 h and 120 h) the cells were washed with 10 mL PBS and harvested by scraping in 20 mL PBS. Cells were pelleted by centrifugation at 800 g for 5 min. The cells were lyzed by resuspending in 500 µL lysis buffer and incubation for 10 min on ice. Soluble and insoluble fraction were isolated by centrifugation at 21000 g for 20 min at 5 °C and insoluble fraction was resuspended in 50 µL lysis buffer by sonication under ice cooling. The samples then underwent subsequently *Click reaction and preparative gel-based analysis* (see below).

In situ ABPP labeling experiments

Analytical labeling in PBS: Cells were grown in 6 well plates until a confluency of 70% was reached. The media was exchanged to PBS containing either probes at a concentration of 50 or 100 µM or the corresponding amount of DMSO as control. After incubation with probes for 2 h cells were washed with PBS and harvested by scraping in 1.5 mL PBS. Cells were pelleted by centrifugation at 800 g for 5 min and PBS was removed. Cells were lyzed in

50 µL lysis buffer (PBS, 1% NP-40, 1% DOC) and incubated on ice for 10 min. Soluble and insoluble fractions were separated by centrifugation at 21000 g for 20 min at 5 °C. The insoluble fraction was resuspended in 50 µL lysis buffer by sonication under ice cooling. The samples then underwent *Click reaction and analytic gel-based analysis* (see below).

Analytical labeling in medium: Cells were grown in 6 well plates until 70% confluent. The probes were added to the wells with a dilution of 1:1000 from DMSO stocks and the control well was supplemented with the corresponding amount of DMSO. After incubation for 3.5 h cells were washed with PBS and harvested by scraping in 1.5 mL PBS. Cells were pelleted by centrifugation at 800 g for 5 min and PBS was removed. Cells were lyzed by resuspension in 50 µL lysis buffer (PBS, 1% NP-40, 1% DOC) and incubation on ice for 10 min. Soluble and insoluble fraction were isolated by centrifugation at 21000 g for 20 min at 5 °C and insoluble fraction was resuspended in 50 µL lysis buffer by sonication under ice cooling. The samples then underwent *Click reaction and analytic gel-based analysis* (see below).

Preparative labeling: Cells were grown to 70% confluency in Petri dishes (150 mm). Then the medium was aspirated and cells were washed with 10 mL PBS and then harvested in 20 mL fresh PBS by scraping. Cells were washed (800 g, 5 min), resuspended in 1000 µL PBS containing probes at the appropriate concentration and incubated for 2 h at RT. Subsequently, cells were pelleted for 5 min at 800 g at RT to remove PBS with excess of the probe, washed twice with 500 µL PBS and resuspended in 500 µL lysis buffer. Soluble and insoluble fraction were separated by centrifugation at 21000 g for 60 min at 4 °C. Insoluble pellets were resuspended in 500 µL lysis buffer by sonication under ice cooling. Protein concentration was assayed (Rotiquant universal, *Carl Roth Laborbedarf*) and adjusted to 2 mg/mL in PBS.

Preparative SILAC labeling

In situ: For preparative SILAC labeling cells were grown in media containing a combination of medium (L-[U-13C6,14N4]arginine and L-[2H4]lysine) or heavy (L-[U-13C6,15N4]arginine and L-[U-13C6,15N2]lysine) isotope-labeled forms of arginine and lysine until full incorporation of the amino acids. The further workflow followed the protocol described in the section *Preparative in situ ABPP labeling experiments.* The cell population containing medium isotope-labeled arginine and lysine served as control cells while the cell population containing heavy isotope-labeled forms of the mentioned amino acids underwent treatment with probes. After cell lysis the protein concentration was determined and 2 mg of each SILAC labeled proteome mixed. The total volume was adjusted to

947 µL. The samples underwent *Click reaction and preparative gel-based analysis* (see manuscript).

Five day labeling: Cells grown in media substituted with medium-labeled isotopes of arginine and lysine served as controls and cells grown in media substituted with heavy-labeled isotopes of arginine and lysine were incubated with probe. Incubation and harvest was done according to *five day labeling (preparative)* (see above). After lysis 2 mg of each cell population was mixed together and adjusted to a total volume of 947 µL. The samples underwent *Click reaction and preparative gel-based analysis* (see manuscript).

Competitive control labeling: For competitive labeling, applying the same labeling scheme as above, cells serving as controls were incubated for 15 min with 100 µM acivicin and afterwards 100 µM ACV1 was added and incubated for additional 2 h. The complementary cells were solely incubated with 100 µM ACV1 for 2 h. After incubation lysis was executed according to *Preparative in situ ABPP labeling experiments* and subsequently underwent *Click reaction and preparative gel-based analysis* (see manuscript) and 50 µL of lysates underwent *Click reaction and analytic gel-based analysis* (see below). The experiment was repeated once maintaining the same labeling scheme of SILAC cell labels and probe treatment. A third experiment was carried out with pre-treatment of heavy labeled cells with 100 µM acivicin for 15 min following incubation with 100 µM ACV1 for 2 h and treatment of medium labeled cells with 100 µM ACV1 for 2 h.

Click reaction and analytical gel-based analysis

To 45 µL of proteome 1 µL RhN_3 (5 mM in DMSO) was added, followed by 1 µL TCEP solution (53 mM in ddH_2O) and 3 µL ligand TBTA (83 mM in DMSO/*tert*-butanol). Samples were gently vortexed and the cycloaddition was initiated by the addition of 1 µL $CuSO_4$ solution (50 mM in ddH_2O). The reaction was incubated for 1 h at RT. For analytical gel electrophoresis, 50 µL 2xSDS loading buffer were added and 50 µL were applied on the gel. Roti®-Mark STANDARD (*Carl Roth GmbH & Co. KG*, for Coomassie staining) and BenchMark™ Fluorescent Protein Standard (*Life Technologies*) were applied as markers to determine the protein mass. Fluorescence scans of SDS gels were performed with a *Fujifilm* Las-4000 luminescent image analyser containing a VRF43LMD3 lens and a 575DF20 filter.

Mouse liver lysate

300 mg of mouse liver were cut into smaller pieces and added to a 2 mL tube containing ceramic beads (91-PCS-CKM, *peqlab*) and 1 mL cold PBS was added. The liver was homogenized by a Precellys 24 homogenizer (*peqlab*) with 5000 rpm for 10 sec in two cycles.

Soluble and insoluble fraction were separated by spinning at 21000 g for 20 min at 5 °C. The insoluble pellet was resuspended in 1 mL PBS by sonication. Protein concentration was assayed (Rotiquant universal, *Carl Roth Laborbedarf*) and adjusted to 2 mg/mL in PBS.

In vitro labeling experiments

Analytical: 1 µl probe stock was added to 44 µL proteome to adjust the desired concentration and incubated at RT for 1 h. After incubation *Click reaction and analytical gel-based analysis* (see above) was performed.

Preparative: 1 µL probe stock was added to 946 µL proteome solution to get the appropriate probe concentration (50 µM for ACVL probes and 100 µM ACV2 and 20 µM ACV1) with one sample lacking the probe as control. The solution was incubated for 1 h at RT. Subsequently *Click reaction and preparative gel-based analysis* (see above) was performed.

Competitive labeling experiments

43 uL of proteome solution were incubated with 1 µL of acivicin stock to adjust the desired concentration (see gel below) and incubated for 15 min at RT. After pre-incubation 1 µL of probe stock was added to adjust the desired probe concentration and incubated for 1 h at RT. After that *Click reaction and analytical gel-based analysis* (see above) was performed.

Labeling of recombinant CES1

1 µL of CES1 stock (5 µg/µL) was added to 43 µL PBS or 43 µL A549 lysate. One sample containing 44 µL PBS or 44 µL A549 lysate was incubated with 1 µL DMSO and served as control. The heat control consisted of 43 µL PBS or 43 µL A549 lysate with 1 µL CES1 stock (5 µg/µL) and was treated for 7 min at 96 °C. The samples were incubated with 1 µL probe stock to obtain a concentration of 10 µM FP-probe and 20 µM for ACV2 probe for 1 h at RT. After completion *Click reaction and analytical gel-based analysis* (see above) was performed.

Labeling of recombinant CES1 for 24 h

Three samples were prepared by adding 1 µL of CES1 stock (5 µg/µL) to 43 µl PBS and two samples by adding 1 µL CES1 stock to 43 µL A549 lysate. One sample of each set served as control lacking probe and was incubated with 1 µL DMSO. Another sample from each set was incubated with ACV2 at a concentration of 20 µM and incubated for 24 h at RT. As a positive control one sample of CES1 in PBS was labeled with 10 µM FP probe for 1 h at RT. After completion of incubation *Click reaction and analytical gel-based analysis* (see above) was performed.

ALDH-Assay

For inhibition assays, 1 µl of probe stock with different concentrations was pre-incubated with 3.5 µg ALDH1A1 or ALDH4A1 in 49 µL 50 mM Tris-HCl (pH 8.5) for 30 min at RT. Afterwards, 50 µL substrate mixture containing 5 mM Tris-HCl, 100 mM KCl, 5 mM β-mercaptoethanol, 1 mM β-NAD^+ and 100 µM propionaldehyde were added to initiate the enzymatic reaction. The product formation of NADH was monitored by measuring the absorption increase at 340 nm at 37 °C in flat bottom 96-well-plates with a *TECAN* Infinite 200pro plate reader. Uninhibited ALDH1A1 served as control. All measurements were carried out as triplicates in at least three independent experiments. Error bars were calculated from standard deviation from the mean. EC_{50} values were calculated from curve fittings by Origin Pro 8.5 (*OriginLab Corporation*).

CES Assay

In a 96 flat bottom well plate 2.5 µg CES1 (*Sigma Aldrich*) in 49 µL 100 mM sodium phosphate (pH 7.4) was pre-incubated with 1 µl probe stock, in different concentrations or DMSO as control, for different times (1 h, 2 h, 3 h, 4 h). After pre-incubation 50 µL 1 mM 4-nitrophenyl acetate in 100 mM sodium phosphate (pH 7.4) was added and the reaction was monitored at 410 nm, 37 °C for 20 min using a *TECAN* Infinite 200pro plate reader. All measurements were carried out in triplicates and in two independent trials. Error bars represent standard deviation from the mean.

Western Blotting

For ALDH4A1 and CES1 detection, the cell lysate samples were prepared in the identical way as for in-gel fluorescence scanning. The proteins separated by 10% SDS-PAGE were transferred to a PVDF membrane (*Biorad*) with a semi-dry blotter (*Biorad*) for 45 min at 12 V. The blots were saturated with 5% non-fat dried milk in PBS-T (PBS with 0.05% Tween 20®, pH 7.4) for 1 h at RT, washed three times with PBS-T for 15 min and incubated with primary rabbit polyclonal anti-CES1 antibody (*Abnova*, 1:5000 dilution in 5% milk in PBS-T) or rabbit polyclonal anti-ALDH4A1 antibody (*Abcam*, 1:5000 dilution in 5% milk in PBS-T) at 4 °C overnight, washed three times for 15 min with PBS-T and detected with goat anti-rabbit IgG HRP-conjugated secondary antibody (*Thermo Fisher Scientific*, 1:10000 dilution in 5% milk in PBS-T, 1 h at RT) and afterwards washed three times with PBS-T for 15 min. Signals were detected using *Amersham* ECL Plus Western Blotting Detection reagents (*GE Healthcare*) using a *Fujifilm* Las-4000 luminescent image analyser containing a VRF43LMD3 lens with no filter. For actin detection membrane was stripped by incubating in 62.5 mM TrisHCl, 100 mM ß-mercapto-Ethanol, 2% SDS, pH 6.8 for 40 min at 52 °C, followed by 10 washes with PBS-T for 15 min. The blots were blocked with 5% non-fat dried milk in PBS-T for 1 h at RT, washed three times with PBS-T for 15 min and incubated with primary goat polyclonal anti-actin antibody (*SCBT*, 1:5000 dilution in 5% milk in PBS-T) at 4 °C overnight. After incubation the blots were washed three times for 15 min with PBS-T and incubated with donkey anti-goat IgG HRP-conjugated secondary antibody (*SCBT*, 1:10000 dilution in 5% milk in PBS-T, 1 h at RT), washed three times with PBS-T for 15 min and detected as described above.

Expression level determination

HepG2 cells were cultivated in 6 wells until 70% confluency. Media was exchanged and 25 µM ACV1 and 10 µM ACV2 were added 1:1000 from DMSO stocks and one 1 mL media was supplemented with 1 µL DMSO as control. After incubation for DMSO control (24 h) and for probes (24 h, 48 h, 72 h, 96 h and 120 h) cells were harvested as described above and lyzed using lysis buffer. Protein concentration was determined using BCA (Rotiquant universal, *Carl Roth Laborbedarf*) and 60 µg of proteome underwent *Western blotting* (described above).

Cloning

To validate the protein hits of the ABPP approach that were identified by MS, we recombinantly expressed these proteins in *E. coli* using the *Invitrogen*™ Gateway® Technology. cDNA of genes ALDH1B1, ALDH2 and ACAA2 were purchased from *Gene Copeia*, ALDH4A1 cDNA was purchased from *Capital Biosciences*. ALDH1A1 cDNA was prepared as described previously [4]. Gene CES1 was synthesized by Life technologies.

Genes ALDH2, ALDH1B1, ALDH4A1 were available in a pDONR™ vector system and were cloned into the pDEST 007 vector using the LR Clonase™ II enzyme mix according to the manufacturer's instructions. The expression clone was transformed in chemically competent SoluBL21™ Competent *E. coli* cells (*Amsbio*) and selected on LB agar plates containing 100 µg mL^{-1} ampicillin. Cloning results were validated after plasmid amplification and preparation (*E.Z.N.A.*™ Plasmid Mini Kit) by sequencing.

CES1 was recombined into pT-Rex™-DEST30 Vector (*Invitrogen*) by LR Clonase™ II enzyme mix in TE buffer according to manufacturer's conditions. The expression clone was transformed in chemically competent One Shot® TOP10 *E. coli* (*Invitrogen*) and selected on LB agar plates containing 100 µg mL^{-1} ampicillin and grown in ampicillin LB. Cells were harvested and plasmids were isolated using a *QIAGEN* Plasmid Midi Kit. Validity of the clones was confirmed by plasmid sequence analysis.

Overexpression for target validation

Expression clones ALDH1A1, ALDH1B1, ALDH2, ALDH4A1 and ACAA2 were grown in 10 mL ampicillin LB at 37 °C with two cultures for each gene until OD_{600} of 0.5 and subsequently one sample was induced with anhydrotetracycline and cultivated for additional 3 h the corresponding sample was cultivated the additional 3 h without induction. The bacterial cell pellets were washed with PBS and resuspended in 100 µL PBS containing 50 µM ACVL1 for ALDH1A1, ALDH1B1, ALDH2; 50 µM ACVL2a for ACAA2 and 50 µM ACV1 for ALDH4A1. The cells were incubated for 2 h at RT and lyzed by sonication. Soluble and insoluble fraction were separated by centrifugation (21000 g for 20 min at 5 °C). Insoluble pellets were resuspended in 100 µL PBS using sonication. The samples underwent *Click reaction and analytical gel-based analysis* (see above).

Overexpression and purification of ALDH4A1

Cells were grown in ampicillin M9 media at 37 °C until an OD600 of 0.7, then induced with anhydrotetracycline 1:20000 (200 ng/µL) and cultivated for 20 h at 20 °C. Bacteria were

harvested by centrifugation (5000 g for 20 min), cell pellets were washed with PBS, resuspended in binding buffer (100 mM Tris-HCl pH 8.0, 150 mM NaCl, 1 mM EDTA) and lyzed by French press. Cell debris was separated by centrifugation (20000 g 20 min) and the protein was purified with StrepTrap™ HP columns (*GE Healthcare*) and eluted with 100 mM Tris-HCl pH 8.0, 150 mM NaCl, 1 mM EDTA, 2.5 mM desthiobiotin. After exchanging the buffer to 50 mM Tris, 50 mM NaCl, 5% glycerol protein concentrations were determined by BCA assay (Rotiquant universal, *Carl Roth Laborbedarf*).

Overexpression and purification of ALDH1A1

ALDH1A1 was purified as described previously.[4]

Metabolic labeling

In a 6 well plate with HEK293T cells, 4 wells were transfected with pT-Rex™-DEST30 containing the CES1 insert and one well with pT-Rex™-DEST30 Vector (as empty vector control) using Lipofectamine® 2000 according to manufacturer's protocol. In addition in one well HEK293T cells were only treated with transfection reagent. 24 h after transfection, the media of the two control wells and one CES1 transfection well (*in situ*) was exchanged by media containing 25 µM ACV2. The remaining 2 wells transfected with CES1 were exchanged by media without probe. After an incubation of 48 h cells were harvested by scraping and centrifugation (800 g, 5 min), and lyzed by incubation with 50 µL lysis buffer for 10 min on ice. Soluble and insoluble fraction were isolated by centrifugation at 21000 g for 20 min at 5 °C and insoluble fraction was resuspended in 50 µL lysis buffer by sonication under ice cooling. One of the unlabeled CES1 transfected cells was incubated with 25 µM ACV2 for 1 h at RT after cell lysis (*in vitro*). The samples underwent *Click reaction and analytical gel-based analysis* (see above).

Mutagenesis of CES1 and ALDH4A1

To analyze the binding site of ACV2 in CES1 the active site serine was converted to alanine by mutagenesis using the following primers to create the mutant S221A:
Forward primer: 5'-ctttctcctcccgctgcctctccaaagatggtc-3'

Reverse primer: 5'-gaccatctttggagaggcagcgggaggagaaag-3'

For ALDH4A1 the nucleophilic cysteine was exchanged by alanine to generate the mutant C348A:

Forward primer: 5'-cgagcacgcggaagccttctggccaccg-3'

Reverse primer: 5'-cggtggccagaaggcttccgcgtgctcg-3'

The point mutations were incorporated by PCR on the corresponding plasmid created before (CES1 on pT-Rex™-DEST30 vector and ALDH4A1 on pDEST 007 vector). After PCR a DpnI digest was performed according to manufacturer's instructions and plasmids were transformed and amplified in chemically competent *E. coli* XL1 as described above.

Labeling of CES1 and mutant S221A

In a 6 well plate with HEK293T cells two wells were transfected with CES1(wt) and one well withS221A using Lipofectamine® 2000 as described above. 24 h after transfection one well transfected with CES1, one well transfected with S221A and one well containing only HEK293T cells were incubated with 25 µM ACV2. 3 days after transfection, cells were harvested and lyzed as described above. One untreated CES1 transfection was incubated with 10 µM diisopropyl fluorophosphate (DFP) for 15 min and subsequently incubated with 10 µM FP probe. Samples underwent *Click reaction analytical gel-based analysis and western blotting* (see above).

Labeling of ALDH4A1 and mutant C348A

pDEST 007 plasmid carrying ALDH4A1 (wt) and C348A were transformed into competent SoluBL21™ Competent *E. coli* cells (*Amsbio*) and overexpressed as described above (*Overexpression for target validation*). One sample for ALDH4A1 and C348A was not induced and served as a control and one sample overexpressing ALDH4A1 was incubated after lysis with 50 µM disulfiram 15 min prior to incubation with 50 µM ACV1. After incubation *Click reaction analytical gel-based analysis and Western blotting* (see above).

siRNA knockdown

Knockdown of CES1 and ALDH4A1 gene expression was performed with silencer select siRNA (*life technologies*, ALDH4A1 #4390624, CES1 #4392420). For determination of cell growth, HepG2 cells were reverse transfected with Lipofectamine® RNAiMax (*life technologies*) following the manufacturer’s instructions in 96 well plates, with one plate for

each time interval (24 h, 48 h, 72 h and 96 h). Cells only treated with transfection reagent served as negative control.
24 h, 48 h, 72 h and 96 h after the transfection, cells were fixed by addition of 12 µL 11% glutaraldehyde and incubation for 30 min. Media was removed and fixed cells were washed 10 times with ddH_2O and dried over night. The fixed cells were stained by adding 100 µL of 0.1% crystal violet solution in water to each well and incubated for 30 min at room temperature. Crystal violet solution was removed and the wells were washed 10 times with ddH_2O and dried over night. The dried stained cells were lyzed by addition of 100 µL 10% acetic acid. The optical density at 590 nm was measured using a *TECAN* Infinite 200pro plate reader. All knockdowns and controls were done five times for each time interval and repeated in at least two independent trials. Error bars were calculated from standard deviation from the mean.
To evaluate knockdown efficiency 40 µg lysate were labeled with 10 µM FP for CES1 and 50 µM ACV1 for ALDH4A1 for 1 h and *Click reaction and analytical gel-based analysis* and Immunoblotting was carried out with actin as loading control (see above).

In gel digestion

Protein bands were excised from the gel and subsequently washed with ddH_2O (100 µL, 15 min, 550 rpm, RT), 50 mM ammonium bicarbonate in MeCN (200 µL, 15 min, 550 rpm, RT) and dehydrated with 100% MeCN (100 µL, 10 min, 550 rpm, RT). The shrunken gel pieces were rehydrated in 50 mM ammonium bicarbonate (100 µL, 5 min, 550 rpm, RT), followed by a further dehydration step (100 µL MeCN, 15 min, 550 rpm, RT). The supernatant was removed and the gel pieces were again washed with MeCN (100 µL, 10 min, 550 rpm, RT) and then dried under vacuum in a centrifugal evaporator (15 min, 1 mbar, RT). The proteins were reduced by addition of DTT solution (10 mM in 50 mM ammonium bicarbonate, 100 µL, 45 min, 550 rpm, 56 °C) to the gel pieces. Then the pieces were washed with MeCN (100 µL, 10 min, 550 rpm, RT), prior to alkylation with iodacetamide solution (55 mM in 50 mM ammonium bicarbonate, 100 µL, 30 min in the dark, 550 rpm, RT). The gel pieces were afterwards washed with MeCN:50 mM ammonium bicarbonate (1:1) (100 µL, 15 min, 550 rpm, RT) and MeCN (100 µL, 10 min, 550 rpm, RT) and dried *in vacuo* in a centrifugal evaporator (15 min, 1 mbar, RT). 100 µL digest solution (0.5 µg trypsin in 100 µL 50 mM ammonium bicarbonate) was added and incubated for 10 min at 4 °C, followed by 37 °C, 300 rpm overnight. The next day the supernatant was transferred into a LoBind tube

(*Eppendorf AG*) and 25 mM ammonium bicarbonate (100 µL, 15 min sonication, RT) was added to the gel pieces. Then MeCN (100 µL) was added and the sample sonicated for 15 min. The supernatant was transferred into the same LoBind tube. 5% FA (100 µL, 15 min sonication, RT) was added to the gel pieces, followed by additional MeCN (100 µL, 15 min sonication, RT). The supernatant was transferred into the same LoBind tube and replaced by MeCN (100 µL, 15 min sonication, RT). The supernatant was transferred into the LoBind tube and the solvent was removed *in vacuo* in a vacuum centrifuge (4 h, 1 mbar, RT). The remaining peptides were stored at -20 °C.[5]

Sample preparation for mass spectrometry

Centrifugal filters (modified Nylon, 0.45 µm, low protein binding, *VWR International, LLC*) were pre-rinsed with ddH_2O (1 x 500 µL, 21000 g, 1 min, RT), 0.5 N NaOH (1 x 500 µL, 21000 g, 1 min, RT), ddH_2O (2 x 500 µL, 21000 g, 1 min, RT) and 1 % FA (1 x 500 µL, 21000 g, 1 min, RT). The peptides were dissolved in 1% FA (20 µL, 15 min sonication, RT) and added to the pre equilibrated filters and centrifuged (21000 g, 1 min, RT). The filtrate was transferred into a vial.

Mass spectrometry and bioinformatics

Measurements were performed using an Orbitrap XL coupled online to an Ultimate 3000 nano HPLC system (*Thermo Fisher Scientific*). Samples were loaded on a trap column and separated on a 15 cm C18 column (2 µm, 100 Å, *Thermo Fisher Scientific Inc.*). For protein identification either the five most intense ions of the full scan were fragmented using CID or a combination of CID of the three most intense ions and HCD of the two most intense ions was performed. The mass spectrometry data were searched using the SEQUEST algorithm against the corresponding database (ipi.human.v3.68.fasta and ipi.mouse.v3.68.fasta) via the Proteome Discoverer Software 1.3 (*Thermo Fisher Scientific Inc.*). Peptides were considered with a minimal mass of 350 Da and a maximal mass of 10,000 Da. Protein *N*-terminal acetylation and oxidation on methionine were added as variable modifications. Carbamidomethylation on cysteine was added as fixed modification. Mass tolerances of the precursor and fragment ions were set to 10 ppm and 0.5 Da. Filters were set to further refine the search results. The Xcorr vs. charge state filter was set to Xcorr values of 1.5, 2.0, 2.25 and 2.5 for charge states +1, +2,

+3 and +4, respectively. The number of different peptides had to be ≥ 2 and the peptide confidence filter was set to at least medium. These filter values are similar to others previously reported for SEQUEST analysis. Xcorr values (Score) of each run, the peptide spectrum matches (PSM) as well as the total number of obtained peptides, unique peptide, ratio from SILAC or precursor ion intensity ratio between labeled and control samples are reported below. Identified proteins from gel slices were defined as true probe targets when the peak area of labeled sample versus unlabeled (control) sample is significantly increased. Intensities of proteins from the same gel slice can be estimated by the score and the number of peptide spectral matches (PSMs). Proteins underlying the fluorescence bands in ABPP approaches posses in addition to an increased peak area a high score and number of PSMs compared to other proteins identified in the same slice.

1. G. C. Rudolf and S. A. Sieber, *Chembiochem : a European journal of chemical biology*, 2013, **14**, 2447-2455.
2. R. Orth, T. Bottcher and S. A. Sieber, *Chemical Communications*, 2010, **46**, 8475-8477.
3. J. Eirich, R. Orth and S. A. Sieber, *Journal of the American Chemical Society*, 2011, **133**, 12144-12153.
4. T. Wirth, G. F. Pestel, V. Ganal, T. Kirmeier, I. Schuberth, T. Rein, L. F. Tietze and S. A. Sieber, *Angewandte Chemie*, 2013, **52**, 6921-6925.
5. S. A. Sieber, S. Niessen, H. S. Hoover and B. F. Cravatt, *Nature chemical biology*, 2006, **2**, 274-281.

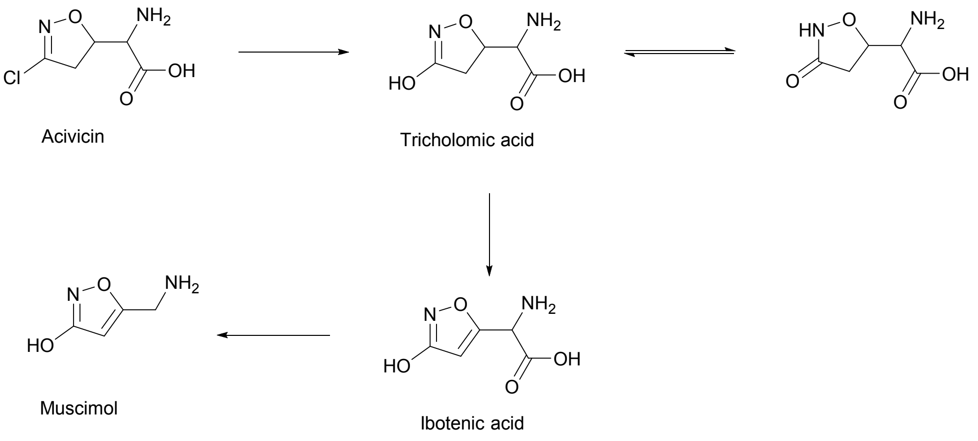

Supporting scheme 1. Possible conversion of acivicin.

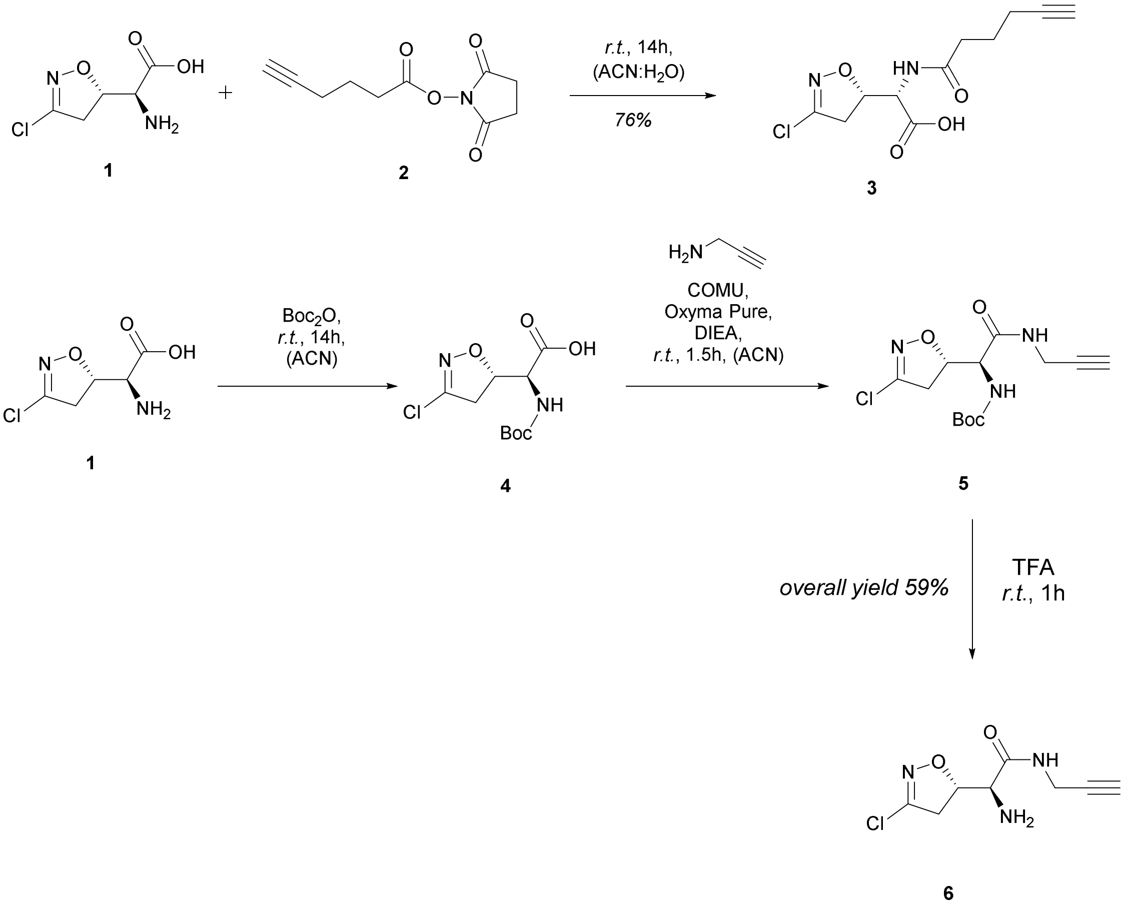

Supporting scheme 2. Synthetic route of ACV1 and ACV2.

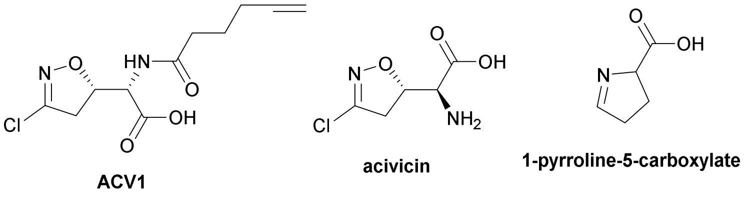

Supporting scheme 3. Structures of the ALDH4A1 inhibitors ACV1 and acivicin and the substrate of ALDH4A1 1-pyrroline-5-carboxylate.

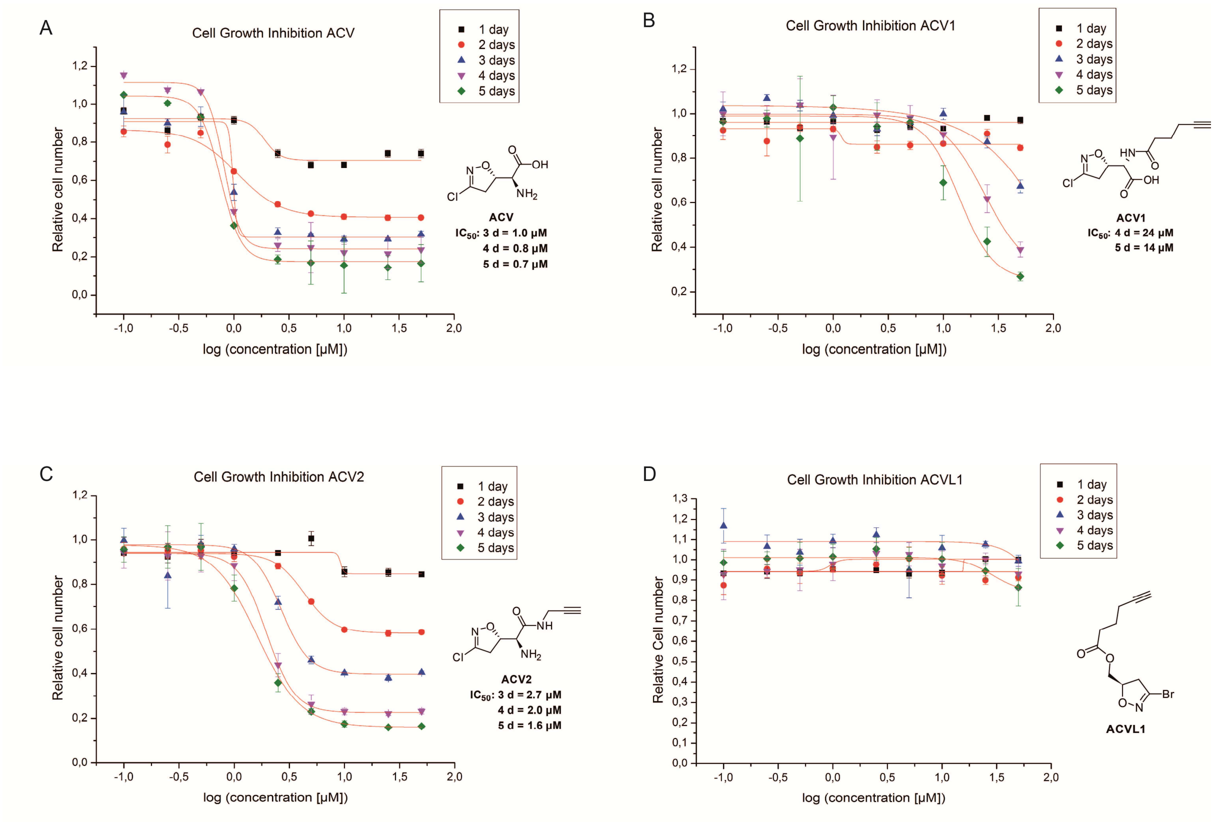

Supporting fig. 1. IC_{50} curves determined from cell growth assay (see Figure 2) for A) ACV, B) ACV1, C) ACV2 and D) ACVL1, that shows no inhibition. Cell number was normalised against control.

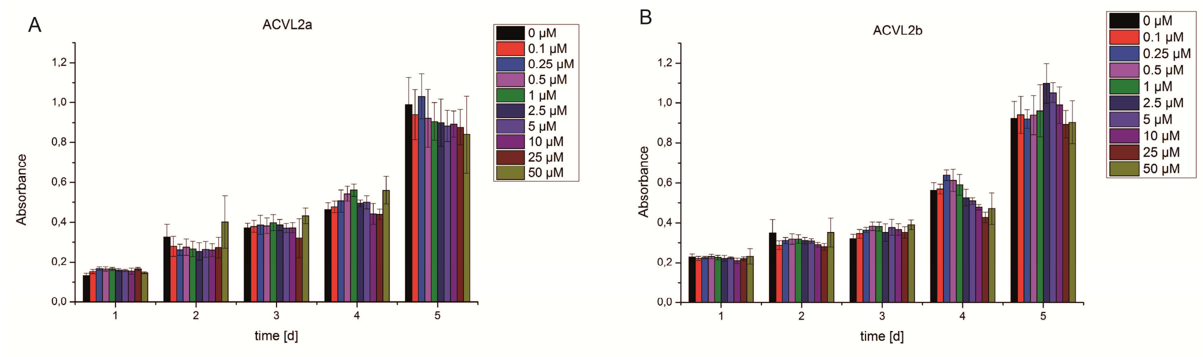

Supporting fig. 2. Cell growth of HepG2 in the presence of ACVL2a and ACVL2b. For each concentration experiments were carried out five times in at least two independent experiments. The error bars display standard deviation from the mean.

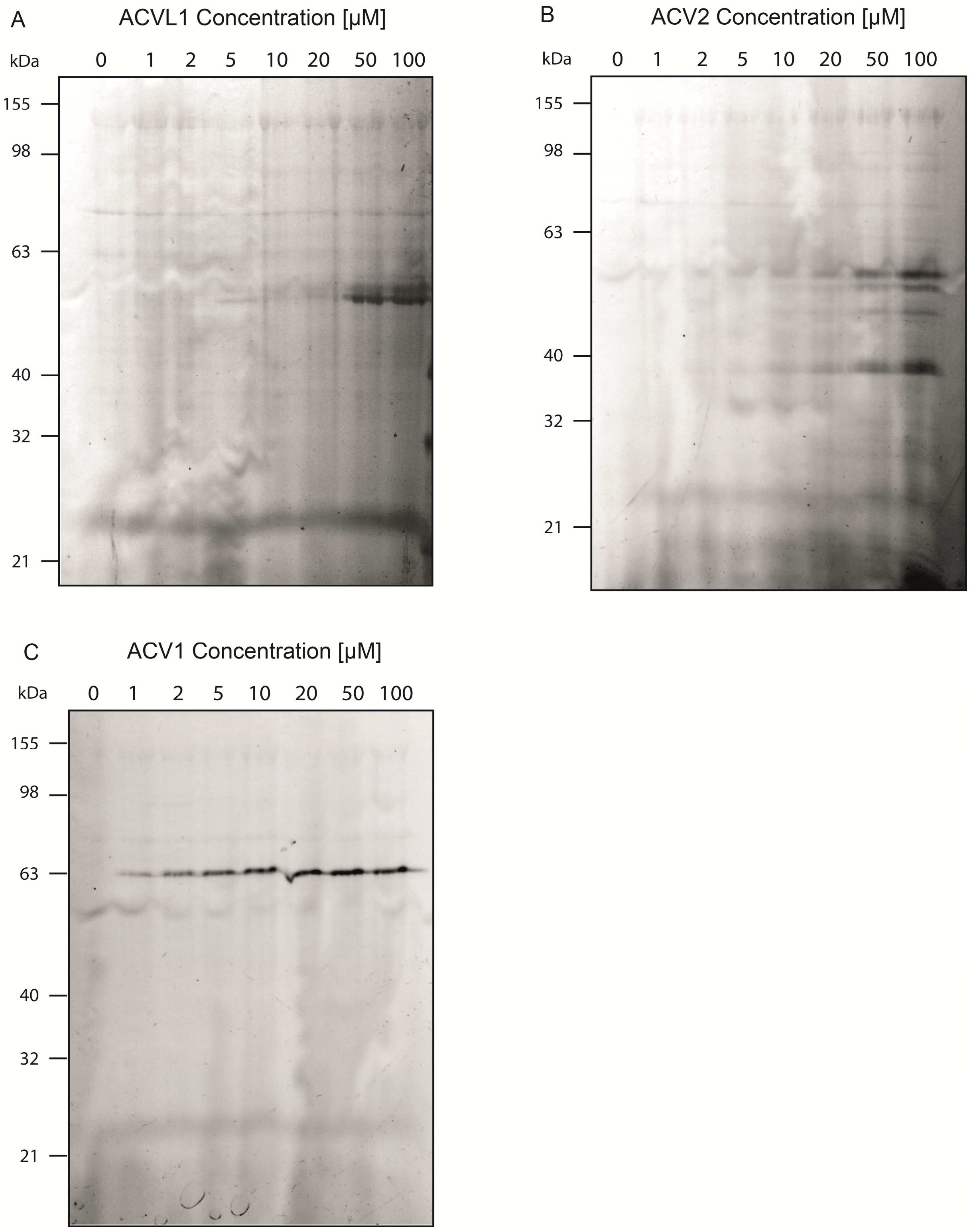

Supporting fig 3. Concentration-dependent activity-based protein profiling of probes A) ACVL1, B) ACV2 and C) ACV1 in mouse liver.

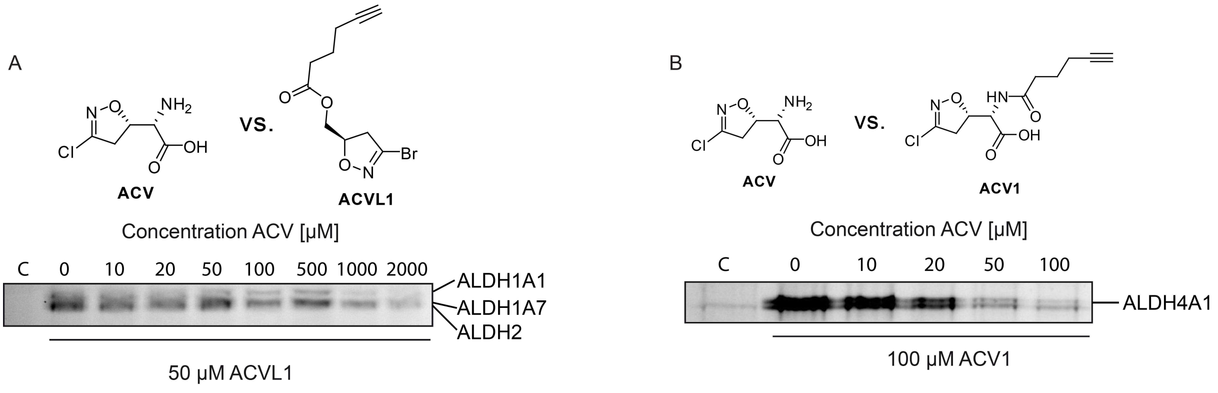

Supporting fig. 4. Competitive labeling in mouseliver of A) acivicin against ACVL1 and B) acivicin against ACV1.

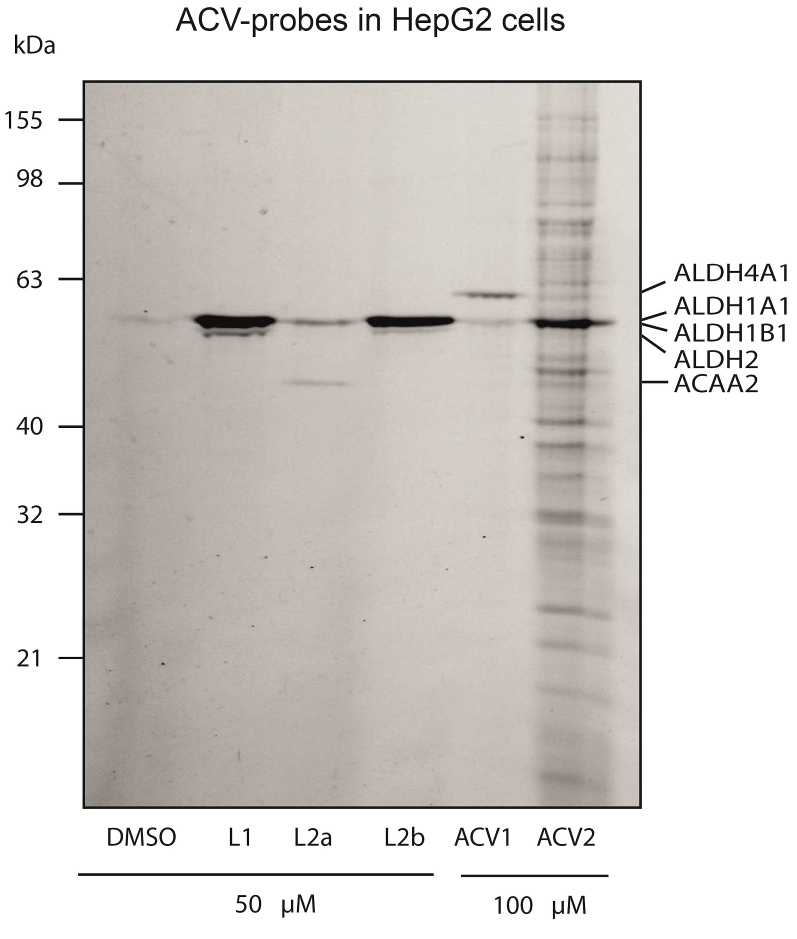

Supporting fig. 5. *In situ* labeling pattern of ACV probe library in living HepG2 cells in PBS. ACVL1 and ACVL2b show labeling of ALDHs 1A1, 2, 1B1. ACVL2a labels ACAA2. ACV1 shows labeling of ALDH4A1. ACV2 shows labeling of ALDH1A1 and ALDH2. For further information of MS results please refer to supporting table 1 and 3.

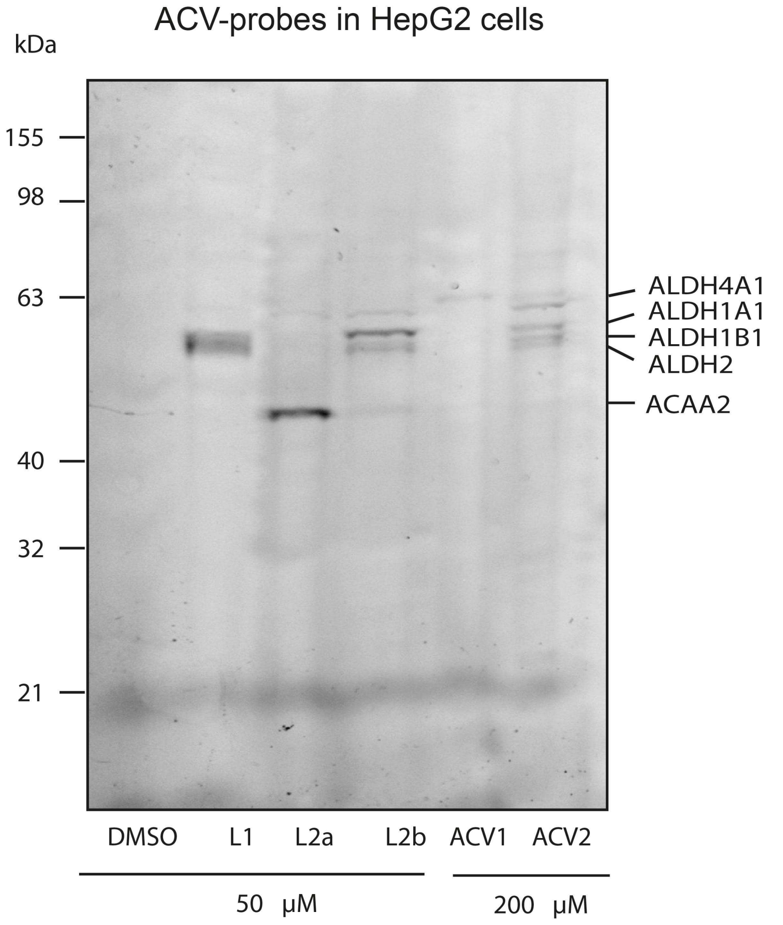

Supporting fig. 6. Labeling of HepG2 cells by ACV-probe library after 3.5 hours incubation in media. ACVL1, ACVL2b and ACV2 show similar labeling preference for ALDHs 1A1, 1B1, 2, compared to labeling in PBS (see above). ACV1 shows preference for ALDH4A1.

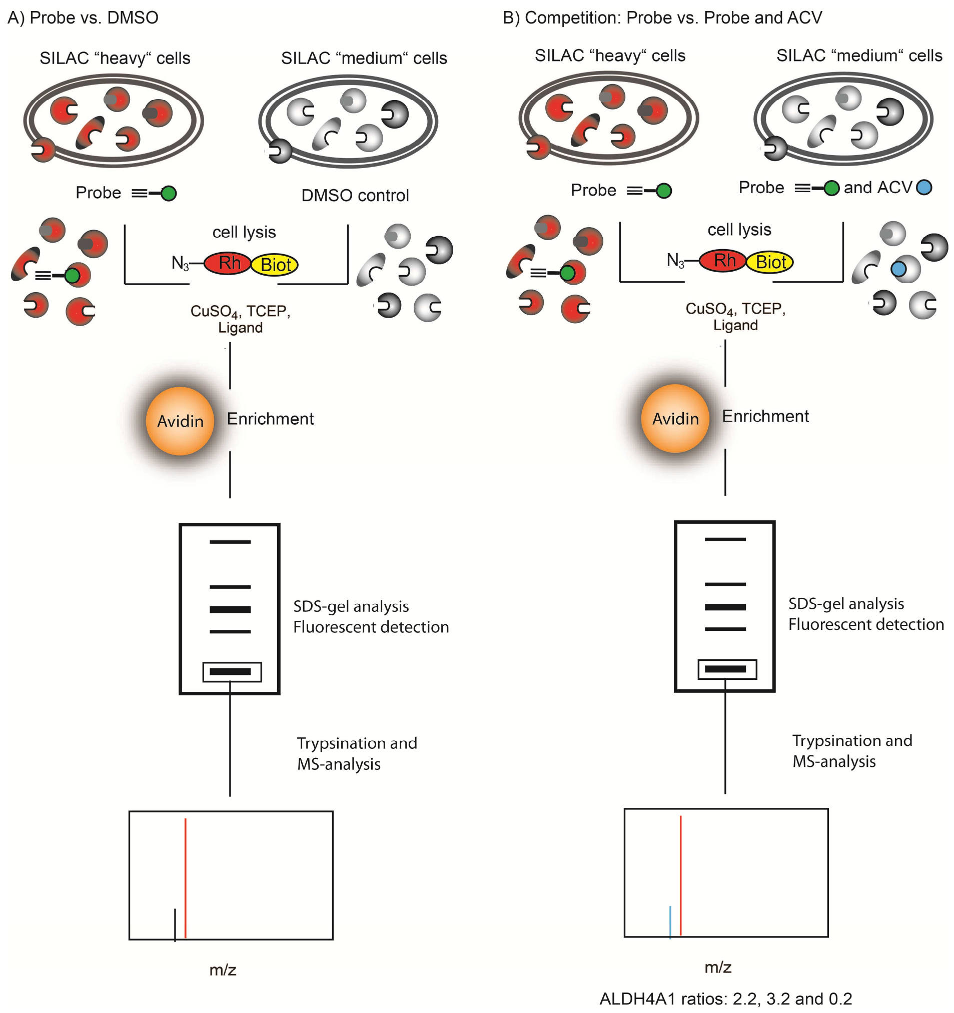

Supporting fig. 7. A) ABPP workflow of SILAC labeled cells with probes and DMSO as control. B) ABPP workflow of SILAC labeled cells with probe (ACV1) and ACV incubated together with probe (ACV1) as control. ALDH4A1 was identified in 3 biological replicates with ratios greater 2, including one SILAC label switch with a ratio below 0.5.

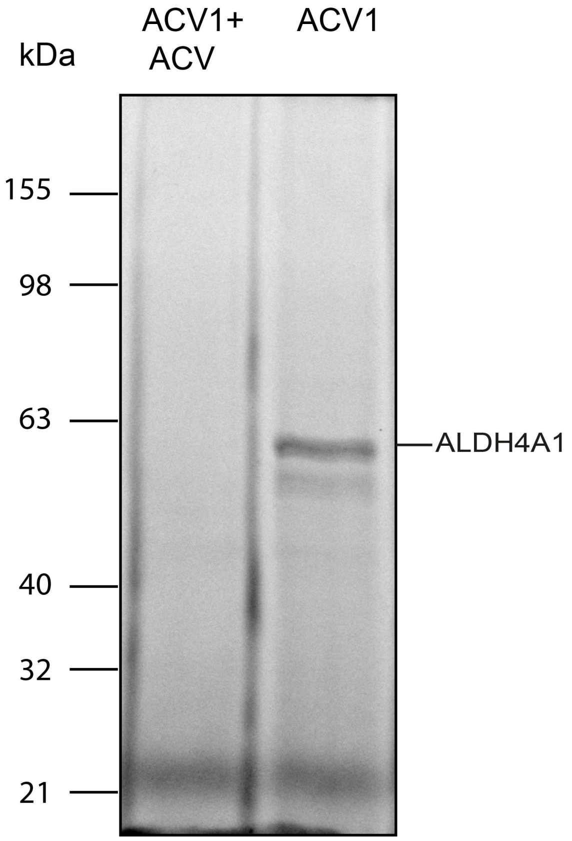

Supporting fig. 8. In situ labeling of intact HepG2 cells with 100 µM ACV1 after 15 minutes pre-incubation with 100 µM acivicin on the left lane and of HepG2 cells incubated with 100 µM ACV1 on the right lane.

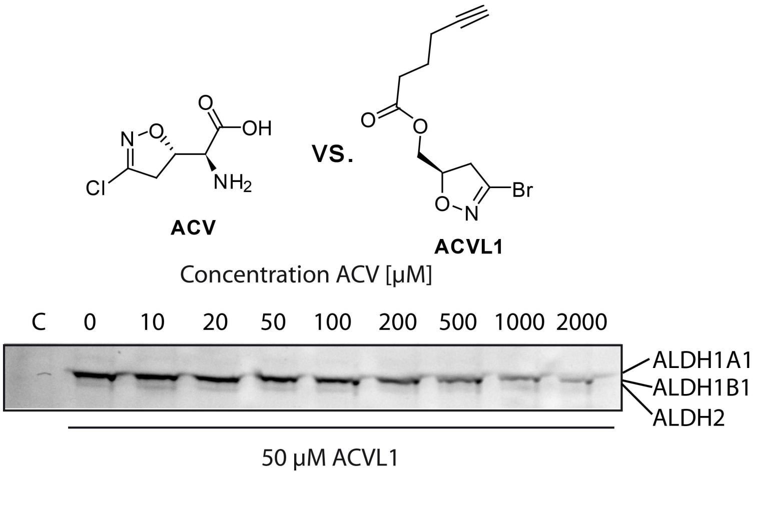

Supporting fig. 9. Competitive labeling in HepG2 lysate of acivicin against ACVL1.

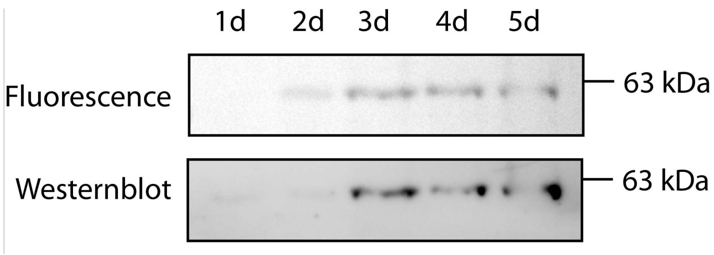

Supporting fig. 10. Confirmation of ALDH4A1 as ACV1 target. HepG2 cells were labeled with ACV1 for up to five days, cell lysates of the different time points were separated by gel electrophoresis and blotted on a PVDF membrane. Detection of the fluorescence signal of the probe (top) and of the specific anti ALDH4A1 antibody (bottom) reveals exactly the same bands.

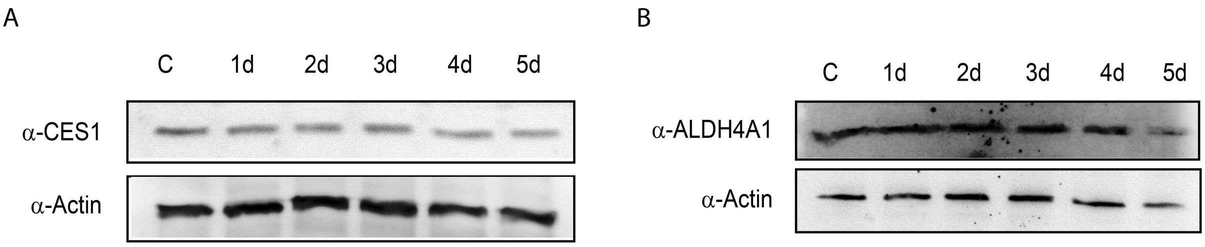

Supporting fig. 11. Western blot analysis of expression level in HepG2 of A) CES1 upon treatment with ACV2 for 1 to 5 days and B) of ALDH4A1 upon treatment with ACV1 for 1 to 5 days. Protein expression levels of the two target proteins are not influenced upon probe treatment.

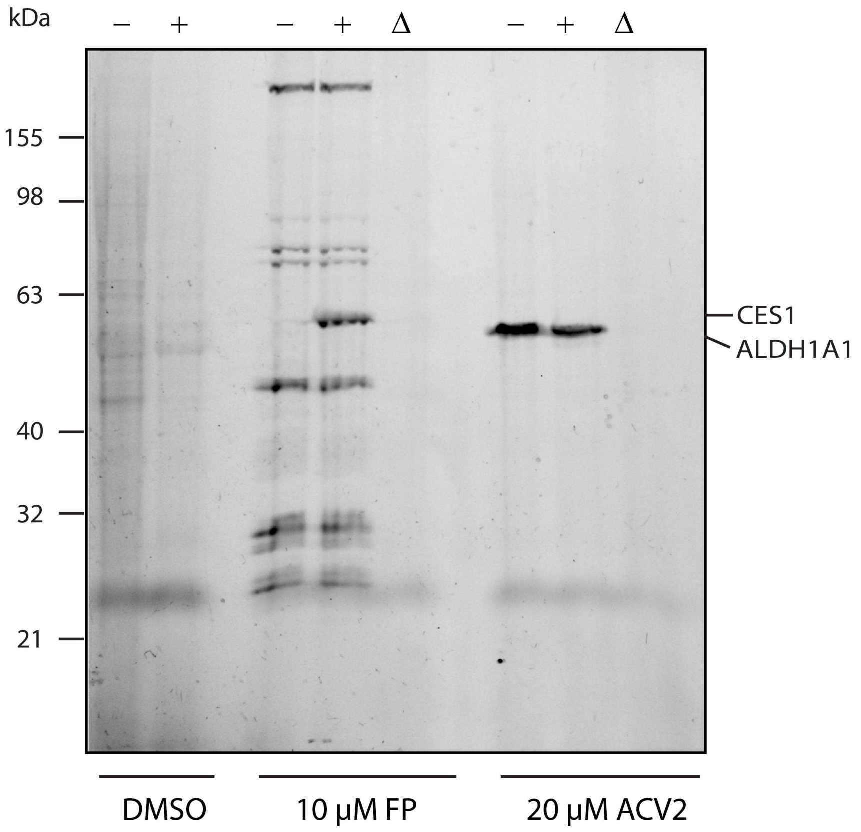

Supporting fig. 12. Labeling of recombinant CES1 in A549 lysate. Incubation with FP showed the expected result with labeled CES1 that was spiked in A549 lysate (+). ACV2 shows no labeling on CES1 but a strong labeling of ALDH1A1 which is below the CES1 signal. The heat control shows no unspecific binding of both probes.

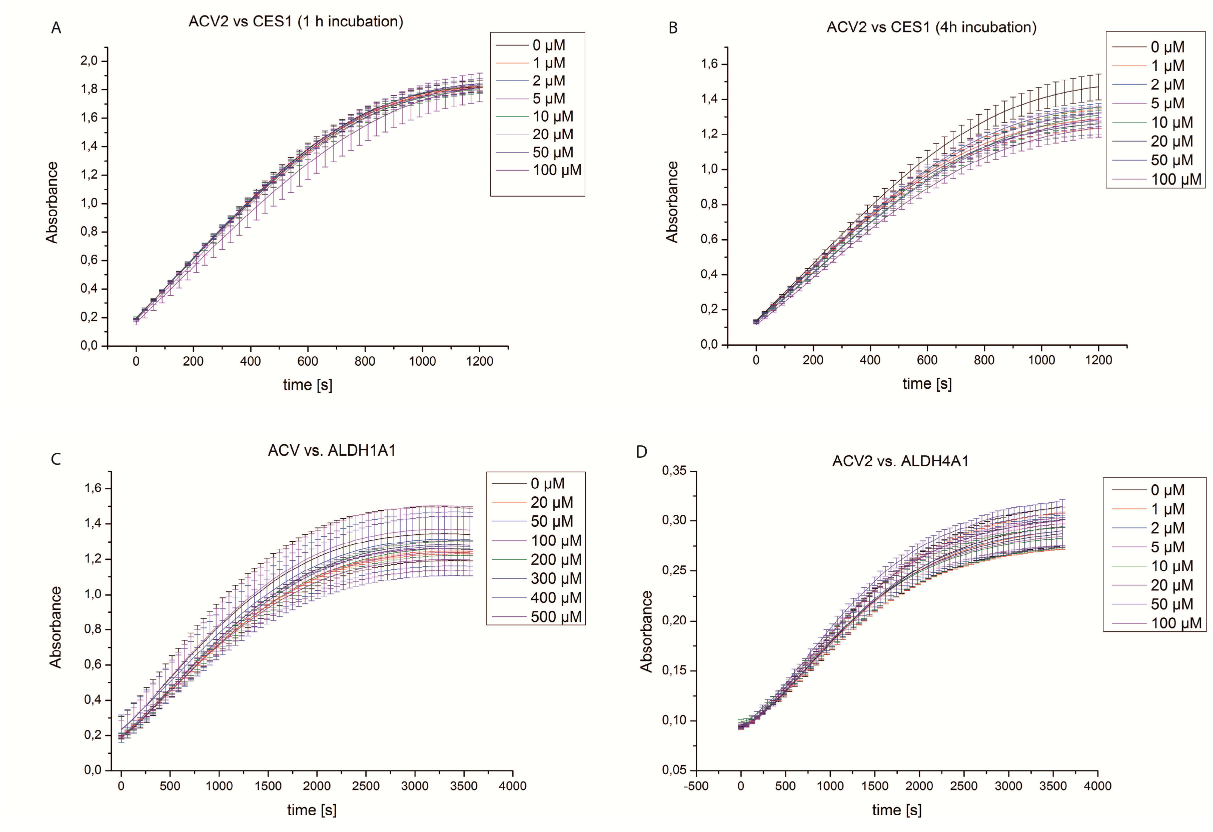

Supporting fig. 13. Conversions of substrates showing no inhibition of the corresponding enzyme. A) The conversion of 4-nitrophenol acetate by CES1 showed no inhibition of CES1 by ACV2 after one hour of pre-incubation and B) only very weak to almost no inhibition of CES1 by ACV2 after extending the pre-incubation to 4 hours can be observed. C) Conversion of propionic aldehyde by ALDH1A1 displayed no inhibition by ACV, even at high concentrations and D) ACV2 showed no inhibition of ALDH4A1.

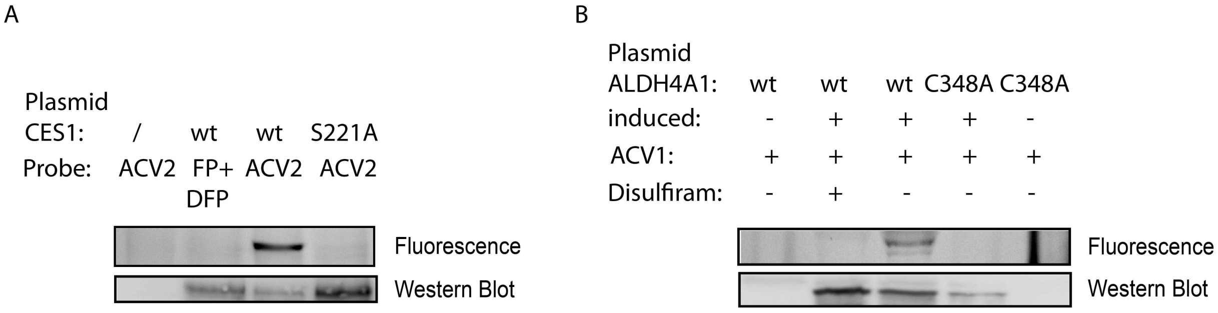

Supporting fig. 14. A) Labeling and western blot analysis of HEK293T cells and HEK293T cells overexpressing CES1 and S221A mutant. ACV2 was incubated for 48 h with both cell lines HEK293T cells and HEK293T cells overexpressing CES1 and in addition S221A mutant. CES1 overexpressed in HEK293T was incubated with 10 µM DFP (diisopropyl

fluorophosphate, a serine protease inhibitor) for 15 min and afterwards with 10 µM FP (fluorophosphonate probe, a serine hydrolase specific probe) (lane 2).

B) Labeling with 50 µM ACV1 and western blot of *E. coli* transformed with expression plasmids of ALDH4A1 and C348A mutant. As negative control *E. coli* containing the respective plasmids were not induced (lanes 1 and 5). For competitive labeling (lane 2), wt ALDH4A1 was incubated with 50 µM disulfiram for 15 min followed by incubation with 50 µM ACV1.

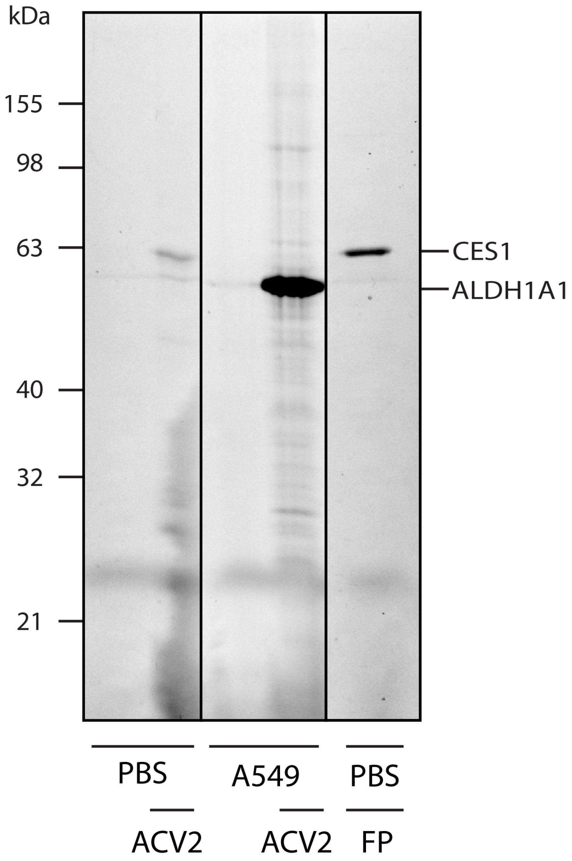

Supporting fig. 15. Labeling of recombinant CES1 in PBS and A549 lysates with incubation for 24 hours. In A549 lysates a strong labeling of ALDH1A1 by 20 µM ACV2 was observed but no labeling of CES1 in comparison to the positive control of CES1 labeled with 10 µM FP. Only weak labeling of CES1 in PBS by 20 µM ACV2 can be observed.

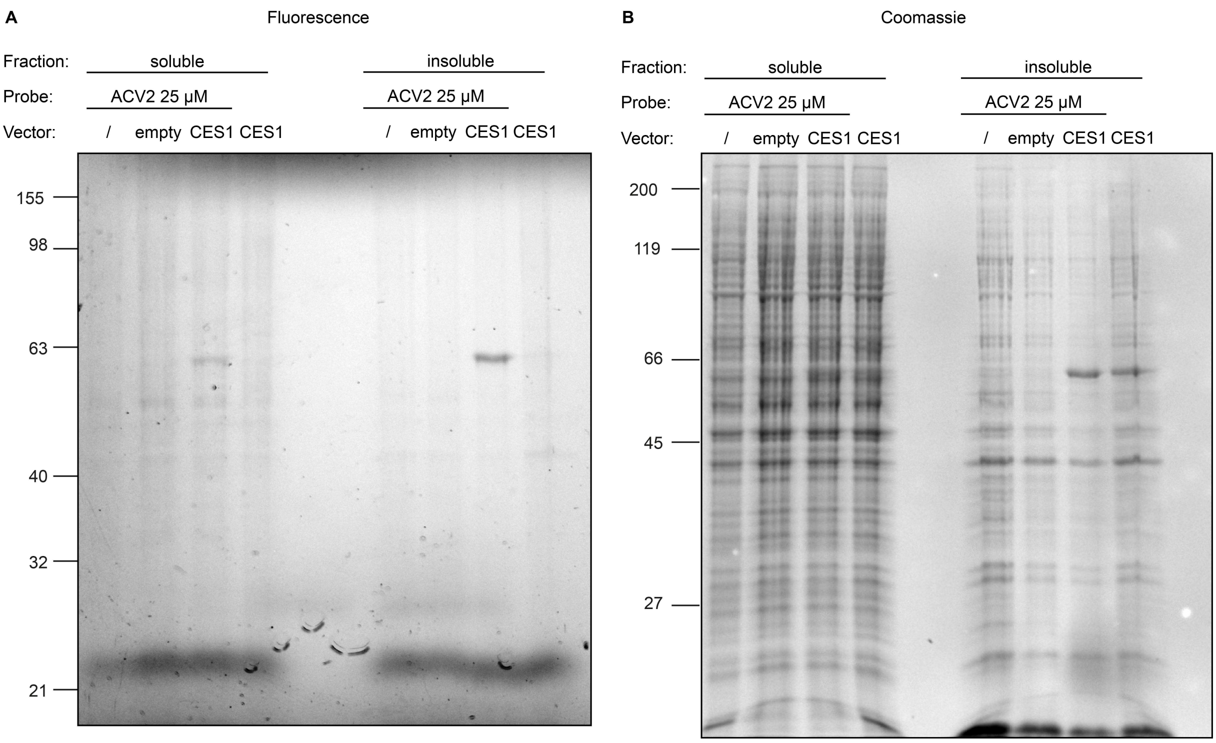

Supporting fig. 16. A) Whole fluorescence gel of metabolic labeling of ACV2 by HEK293T cells overexpressing CES1, with control lacking the plasmid, empty vector control and HEK293T cells overexpressing CES1 without ACV2. B) Corresponding coomassie gel.

Supporting table 1. Overview of protein targets of acvicin derivatives in murine liver tissue and HepG2 cells identified by mass spectrometry. Detailed information of MS results is shown in supporting tables 2 to 6 as pointed out in the table. Underlying criteria for probe target validation is the increase in peak area of probe labeled versus unlabeled (control) sample. Furthermore the score and the number of peptide spectral matches (PSM) clearly define a protein from a gel slice as positive hit. For SILAC experiments ratio of probe versus control defined a clear hit.

Murine liver tissue (Supporting table 2)

Probe	Target	Accession	Description	Area				Control (1)			Control (2)			Probe (1)			Probe (2)			General information		
				Control (1)	Control (2)	Probe (1)	Probe (2)	Score	# Peptides	# PSM	Score	# Peptides	# PSM	Score	# Peptides	# PSM	Score	# Peptides	# PSM	# AAs	MW [kDa]	Sequence coverage
ACVL2a	ACAA2	IPI00226430.2	3-ketoacyl-CoA thiolase, mitochondrial	0,000E0	1,965E8	5,741E8	8,408E8				34,6	7	10	133,1	15	38	67,6	9	21	397	41,8	56,4
ACVL2b	ALDH1A7	IPI00336362.2	Aldehyde dehydrogenase, cytosolic 1	7,571E7	6,715E7	9,488E8	3,334E8	21,5	2	6	7,0	2	2	44,4	11	15	31,3	8	11	501	54,6	39,7
	ALDH2	IPI00111218.1	Aldehyde dehydrogenase, mitochondrial	3,164E7	1,029E8	2,667E9	5,659E9	11,7	2	4	10,7	4	4	117,5	14	39	182,1	20	59	519	56,5	52,4
ACVL2b	ALDH1A1	IPI00626662.3	Retinal dehydrogenase 1	0,000E0	0,000E0	1,409E9	5,290E7							120,3	20	38	5,7	2	2	501	54,4	56,1
ACV1	ALDH4A1	IPI00928176.1	aldehyde dehydrogenase 4A1 precursor	4,052E8	3,589E8	1,893E9	1,655E9	83,6	14	25	97,8	14	29	130,7	13	40	153,4	14	49	562	61,8	39,7
ACV1	ALDH6A1	IPI00461964.3	Methylmalonate-semialdehyde dehydrogenase [acylating], mitochondrial	0,000E0	6,786E8	7,048E8	3,094E9				71,2	13	22	49,3	10	18	237,8	19	71	535	57,9	49,7
ACV2	ALDH1A1	IPI00626662.3	Retinal dehydrogenase 1	2,977E8		5,993E9		90,0	15	29				273,9	25	82				501	54,4	55,7

HepG2 cells labeled in situ for 2 hours (Supporting table 3)

ACVL1	ALDH1A1	IPI00218914.5	Retinal dehydrogenase 1	0,000E0	5,380E7	2,042E9	1,136E10				12,9	4	4	419,2	29	132	652,5	28	208	501	54,8	74,7
ACVL1	ALDH1A1	IPI00218914.5	Retinal dehydrogenase 1	1,341E9	6,403E7	6,630E9	6,114E8	141,5	21	38	30,6	8	9	253,8	25	80	114,2	18	37	501	54,8	63,1
	ALDH2	IPI00006663.1	Aldehyde dehydrogenase, mitochondrial	4,902E8	3,117E8	1,571E10	1,546E9	107,1	17	31	148,6	20	45	916,4	28	284	315,7	22	97	517	56,3	62,5
	ALDH1B1	IPI00103467.5	Aldehyde dehydrogenase X, mitochondrial	4,268E8	0,000E0	1,701E10	4,413E8	90,2	12	25				989,6	23	294	86,9	10	25	517	57,2	44,9
ACVL2a	ACAA2	IPI00001539.8	3-ketoacyl-CoA thiolase, mitochondrial	6,163E8	0,000E0	1,286E9	2,400E8	48,8	11	13				126,7	12	33	100,6	14	26	397	41,9	60,7
ACVL2b	ALDH1A1	IPI00218914.5	Retinal dehydrogenase 1	6,403E7	1,341E9	8,747E8	3,903E9	30,6	8	9	141,5	21	38	176,2	25	54	220,1	26	65	501	54,8	73,1
	ALDH2	IPI00006663.1	Aldehyde dehydrogenase, mitochondrial	3,117E8	4,902E8	1,383E9	1,026E10	148,6	20	45	107,1	17	31	298,0	23	91	793,3	25	264	517	56,3	71,8
ACV1	ALDH4A1	IPI00217871.4	Delta-1-pyrroline-5-carboxylate dehydrogenase, mitochondrial	9,642E7	4,699E7	1,686E10	2,308E9	17,5	5	5	39,9	8	13	664,3	27	215	205,7	19	68	563	61,7	63,6
ACV2	ALDH1A1	IPI00218914.5	Retinal dehydrogenase 1	6,403E7		7,037E8		30,6	8	9				182,2	21	59				501	54,8	58,5
ACV2	ALDH1A1	IPI00218914.5	Retinal dehydrogenase 1	3,536E8	5,380E7	7,037E8	2,824E9	120,4	18	38	12,9	4	4	182,2	21	59	136,9	18	39	501	54,8	61,5
	ALDH2	IPI00006663.1	Aldehyde dehydrogenase, mitochondrial	0,000E0	0,000E0	2,728E8	1,011E9							40,6	10	14	109,7	15	32	517	56,3	45,5

SILAC HepG2 cells labeled in situ for 2 hours (Supporting table 4)

Probe	Target	Accession	Description	Ratio Probe / Control	General information		
					# AAs	MW [kDa]	Sequence coverage
ACVL1	ALDH1A1	IPI00218914.5	Retinal dehydrogenase 1	10,4	501	54,8	41,1
ACVL1	ALDH2	IPI00006663.1	Aldehyde dehydrogenase, mitochondrial	2,6	517	56,3	22,2
	ALDH1B1	IPI00103467.5	Aldehyde dehydrogenase X, mitochondrial	7,8	517	57,2	16,6
ACVL2b	ALDH1A1	IPI00218914.5	Retinal dehydrogenase 1	7,6	501	54,8	21,6
ACV1	ALDH4A1	IPI00217871.4	Delta-1-pyrroline-5-carboxylate dehydrogenase, mitochondrial	2,9	563	61,7	28,2
ACV2	ALDH1A1	IPI00218914.5	Retinal dehydrogenase 1	5,7	501	54,8	41,1

Competitive control experiment in SILAC HepG2 cells (Supporting table 5)

Probe	Target	Accession	Description	1	2	3 (label switch)	General information		
				Ratio ACV1 / (ACV1+ACV)	Ratio ACV1 / (ACV1+ACV)	Ratio (ACV1+ACV) / ACV1	# AAs	MW [kDa]	Sequence coverage
ACV1	ALDH4A1	IPI00217871.4	Delta-1-pyrroline-5-carboxylate dehydrogenase, mitochondrial	2,2	3,2	0,2	563	61,7	33,2

HepG2 cells labeled in situ for 1 to 5 days (Supporting table 6)

Probe	Target	Accession	Description	Area				Control day 1			Control day 5			ACV2 day 1			ACV2 day 5			General information		
				Control day 1	Control day 5	ACV2 day 1	ACV2 day 5	Score	# Peptides	# PSM	Score	# Peptides	# PSM	Score	# Peptides	# PSM	Score	# Peptides	# PSM	# AAs	MW [kDa]	Sequence coverage
ACV2	ALDH1A1	IPI00218914.5	Retinal dehydrogenase 1	1,635E8	2,353E8	4,490E8	5,250E8	70,5	18	23	77,7	15	24	68,9	16	23	92,0	16	31	501	54,8	56,9
	ALDH2	IPI00006663.1	Aldehyde dehydrogenase, mitochondrial	8,303E6	3,508E7	1,654E8	3,199E8	6,3	2	2	22,3	5	8	50,3	14	17	59,6	14	21	517	56,3	38,5
ACV2	CES1	IPI00607693.2	liver carboxylesterase 1 isoform c precursor	1,043E8	4,298E7	1,131E9	1,513E9	39,7	12	12	32,3	10	10	157,4	20	49	249,2	25	80	566	62,4	51,1
